# Unravelling decades of habitat dynamics in protected areas: A hierarchical approach applied to the Gran Paradiso National Park (NW Italy)

**DOI:** 10.1007/s10661-025-14669-0

**Published:** 2025-10-20

**Authors:** Chiara Richiardi, Consolata Siniscalco, Matteo Garbarino, Maria Adamo

**Affiliations:** 1 Laboratory Biodiversity and Ecosystems, Division Anthropic and Climate Change Impacts, Italian National Agency for New Technologies, Energy and Sustainable Economic Development (ENEA), Strada Per Crescentino, Saluggia, VC Italy; 2 Department of Life Sciences and Systems Biology (DBIOS), University of Torino, Viale Pier Andrea Mattioli 25, 10125 Turin, Italy; 3 Department of Agricultural, Forest and Food Sciences (DISAFA), University of Torino, Largo Paolo Braccini 2, 10095 Grugliasco, TO Italy; 4 National Research Council (CNR), Institute of Atmospheric Pollution Research (IIA), c/o Interateneo Physics Department, Via Amendola 173, 70126 Bari, Italy

**Keywords:** Habitat mapping, Vegetation dynamics, Protected area, Mountain area, Monitoring, Remote sensing

## Abstract

Habitat loss is the main threat to biodiversity at a global level, making habitat mapping an essential tool for the management of protected areas and for the conservation and monitoring, in line with Directive 92/43/EEC. Traditional mapping methods are resource-intensive, while remote sensing approaches depend on the availability of ground truth datasets. In this context, this study presents a novel framework for time series habitat classification. The approach leverages a single pre-existing habitat cartography and a limited set of ancillary data to derive a retrospective training dataset. The method was applied to analyse 39 years (1985–2023) of habitat and land cover changes in Gran Paradiso National Park (NW Italy). Annual seasonal composite images were generated for the growing and senescence seasons using an enhanced Best Available Pixel approach. Annually derived training datasets were used to classify hierarchically land cover and habitats via ensemble random forest models. Validation against high-resolution maps demonstrated the robustness of the approach. The method allows for long-term habitat monitoring even in data-sparse environments. The results reveal high stability of land cover (88% of the area) and significant trends in some vegetation types, including a decline in grasslands (− 10 ha year^−1^) and the expansion of shrublands (+ 10 ha year^−1^). The method proved to be reliable for large patches, less so for ecotones. Future research should explore its application across different landscapes. This work underscores the potential of remote sensing for long-term habitat monitoring, providing a cost-effective solution to support biodiversity preservation efforts.

**Supplementary Information:**

The online version contains supplementary material available at 10.1007/s10661-025-14669-0.

## Introduction

Alpine ecosystems, with their peculiar environmental gradients and extreme conditions, host many endemic species and provide key ecosystem services (Grêt-Regamey & Weibel, 2020; Payne et al., 2017). These regions are crucial for both biodiversity conservation and human well-being, including downstream populations in lowland areas where natural ecosystems are often degraded (Ramel et al., 2020). At the same time, alpine areas are particularly vulnerable to the combined effects of climate change and shifting land-use dynamics. Both increased anthropogenic pressure and land abandonment are altering vegetation composition and key biogeochemical processes, with cascading effects on biodiversity and ecosystem service provision (Cannone et al., 2022; Rumpf et al., 2022). Historically, traditional agro-pastoral practices, such as haymaking and grazing, maintained open grasslands and supported high biodiversity. However, since the mid-twentieth century, widespread abandonment of these practices, coupled with climate change, has triggered rapid ecological transitions, leading to the decline of semi-natural grassland habitats (Baur et al., 2006; Lavorel et al., 2017). Given their ecological importance and sensitivity to both land use and climate pressures, focusing on alpine habitats is crucial for assessing conservation status through habitat surface loss dynamics (Ingty, 2021; Yoccoz et al., 2010). Focusing on alpine habitats is therefore not only ecologically justified but also necessary to assess conservation status in light of dynamic surface loss and to understand broader landscape-scale implications for both biodiversity and ecosystem service provision (Ramel et al., 2020; Lavorel et al., 2017). At the same time, alpine areas that are inherently vast and inaccessible can deeply benefit from remote sensing approaches (Abdelmajeed & Juszczak, 2024; Avisse et al., 2017). In this context, long-term habitat mapping can serve as the basis for assessments of biodiversity and ecosystem services, thus supporting environmental planning, management, and preservation (Alvarez-Vanhard et al., 2020; Huber et al., 2022; Kluczek et al., 2023; Price et al., 2023). Remote sensing has transformed habitat mapping by providing synoptic, temporally consistent data across extensive areas (Álvarez-Martínez et al., 2018; Marsoner et al., 2023; Oeser et al., 2020). Compared to traditional field surveys, this approach offers a cost-effective, repeatable, and scalable solution for habitat mapping (Amani et al., 2023; Cruz et al., 2024) and monitoring (Oeser et al., 2020; Praticò et al., 2021). Habitat-level monitoring is also a key requirement under international conservation frameworks, such as the Natura 2000 reporting obligations outlined in the Habitats Directive (Council Directive 92/43/EEC) and the IUCN Red List of Ecosystems, which emphasize the need for consistent, spatially explicit data to assess ecosystem risk status (Dalle Fratte et al., 2019). Earth observation (EO) datasets vary in their spectral, spatial, and temporal features, thus offering different mapping capabilities. Multispectral sensors (e.g., Landsat, Sentinel-2) and hyperspectral imagers (e.g., AVIRIS) deliver fine spectral detail that can resolve vegetation composition, down to species in optimal conditions, and discriminate among forests, grasslands, and wetlands (Amani et al., 2023; Kuenzer et al., 2014; Lahoz-Monfort & Magrath, 2021). Very high resolution (VHR) instruments (e.g., SPOT) capture textures at sub-10 m scales, enabling accurate delineation of patches and small habitats (Zhang et al., 2017). LiDAR adds a third dimension by providing measurements of canopy structure. Synthetic aperture radar (SAR), lastly, complements optical data by operating independently of illumination and cloud cover. SAR backscatter is sensitive to canopy structure and moisture content, which helps distinguish spectrally similar but structurally distinct habitats, particularly in persistently cloudy upland regions (Barrett et al., 2016). However, VHR, LiDAR, and SAR datasets lack the temporal depth required for reconstructing long-term vegetation dynamics. Another limitation for long-term studies is the lack of availability of multi-temporal ground truth datasets. The inherent noise in satellite data archives from constellations such as Landsat limits the applicability of classifiers trained on a single year across multi-temporal datasets (Hansen & Loveland, 2012; Pelletier et al., 2017). This limitation arises from persistent cloud cover that obscures targets, both inter-annual variable snow cover duration and altitude-driven phenological mismatch that complicates the exploitation of phenological information, and radiometric inconsistencies in topographically complex terrains (Heinl & Tappeiner, 2012). In sum, while multi-sensor approaches have greatly enhanced habitat mapping, data gaps, in both space and time, remain a critical barrier to fully characterizing vegetation dynamics. An alternative approach to study vegetation dynamics is the use of trends in vegetation indices, like the normalized difference vegetation index (NDVI) (Carlson et al., 2017a, 2017b; Choler et al., 2021; Filippa et al., 2019a, 2019b; Rumpf et al., 2022), which can serve as a proxy for monitoring vegetation changes, albeit without the ability to differentiate between land cover classes or distinct dynamics, such as grassland decline or shrub encroachment. This lack of granularity limits our understanding of the underlying processes driving these shifts and their broader ecological implications. Addressing these gaps is critical for designing conservation strategies tailored to mitigate biodiversity loss and adapt to rapid environmental changes (Ramskogler et al., 2025).

To address these challenges, we propose a workflow that leverages open source EO imagery, such as Landsat, and robust statistical methods to generate high-resolution, multi-decadal habitat maps. First, we enhance the Landsat archive by applying topographic correction and constructing seasonal Best Available Pixel (BAP) composites. Next, we employ a hierarchical classification scheme: (1) land-cover mapping and (2) within each vegetation class, habitat delineation, via an ensemble of random forest models, using phenological and topographic predictors. A rule-based post-processing filters temporal outliers and isolated misclassifications while enforcing ecological plausibility in habitat trajectories. A key innovation of our workflow is the retrospective application of the Z-statistic, which allows us to infer past habitat distributions from a single reference land cover map (e.g., a cleaned cartography from a known year). Specifically, for each target year, the Z-statistic is used to identify pixels that statistically match the spectral and environmental profile of their assigned class in the reference year. By comparing a pixel’s signature in year T_2_ against the distribution of signatures from its class in the reference year T_1_, we detect and exclude those that likely underwent change. This enables the selection of unchanged, “pure” training pixels across multiple years, even in the absence of multi-temporal ground truth data. The result is a set of temporally consistent training datasets that support the annual reconstruction of habitat maps, facilitating robust long-term change detection and trend analysis. Finally, by quantifying habitat stability and turnover, the workflow identifies persistent versus dynamic areas, informing conservation prioritization and disturbance monitoring. Applied to 39 years (1985–2023) of Landsat data for the Gran Paradiso National Park (Italian Alps), the results demonstrate the utility of scalable, open-data workflows for high-resolution monitoring of alpine ecosystems. Beyond advancing methodological innovation, this study aims to demonstrate how remote sensing and machine learning tools can be operationalized to support conservation practitioners in assessing habitat conditions and tracking vegetation dynamics over time. Strengthening this connection between ecological research and real-world management is essential for translating data-driven insights into effective protection strategies for natural areas.

## Materials and methods

### Study area

Gran Paradiso National Park (Figure S1), Italy’s oldest protected area (established in 1922), spans ~70,000 ha and was created to preserve the Alpine ibex (*Capra ibex*). The park encompasses a wide elevation range (600–4,061 m a.s.l.), hosting diverse ecosystems: broadleaved and mixed forests at lower elevations, coniferous woodlands around mid-altitudes, and subalpine to alpine meadows, mosses, and lichens at higher elevations. The climate is predominantly alpine (ET), with long, snowy winters and short, mild summers. Average winter temperatures often stay below 0 °C, while summer temperatures in the valleys range between 15 and 20 °C. Precipitation is evenly distributed, peaking in late spring and early summer.

### Satellite data and pre-processing

We collected 437 multispectral Landsat scenes (missions 4–9, Collection 2 Level 2, Tier 1) from the USGS Earth Explorer portal, along path 195, row 28, from 1985 to 2023 at 30-m resolution, split into the growing season (15 June–31 August) and the senescence season (15 September–30 November) to capture vegetation phenological shifts. All scenes underwent “improved cosine” terrain correction (Riaño et al., 2003) adapted from the R “*landsat*” package (Goslee, 2012), and cloud/snow masks were generated (Richiardi et al., 2021). To reduce the influence of radiometric distortions due to haze and improve computational efficiency, scenes with cloud cover exceeding 70% were discarded. This threshold was chosen heuristically, yielding a final dataset of 359 scenes. The full list of Landsat scene IDs used in the analysis is available in Supplementary Table S9. The cloud/snow masks fed into the intra-annual Best Available Pixels (BAP) compositing, which uses a six-parameter scoring system (Table S1), following methods proposed by White et al. (2014) and Griffiths et al. (2019). The BAP step was applied to reduce the impact of clouds and other noise and was adapted to alpine environments to also account for snow. Two criteria were adjusted: (i) percentage of snow-free pixels, newly introduced to prioritize dates with minimal snow cover, favoring advanced phenological stages post-snowmelt during the growing season, and snow-free conditions during senescence; and (ii) distance to clouds and snow, modified to favor pixels distant from both clouds (for better radiometric quality) and snow, i.e., selecting pixels further along in phenology during the growing season, and not yet affected by early snowfall in the senescence season. Further details can be found in the cited publications. At the end of this process, we obtained two composite images, one of the growing seasons and one of senescence, for each year, which were then used to compute a series of spectral indices useful to characterize the vegetation, the water, and the soil presence on the ground. The full list of the indices is reported in the Supplementary materials (Table S2). All analyses were conducted within the R environment (R Core Team, 2022).

### Ancillary data

Digital terrain models (DTMs) were acquired from regional geoportals (https://geoportale.regione.vda.it/; https://geoportale.igr.piemonte.it/cms/, accessed on 20/08/2023) and used to derive various topographic variables (Table S2). From the digital surface models (DSMs), obtained from the same sources, we generated the canopy height model (CHM). Both DTMs and DSMs were derived from the same LiDAR flights, performed between 2005 and 2008 for Aosta Valley, and between 2009 and 2011 for Piedmont. Additionally, we used a geological map (https://gn.mase.gov.it/portale/home, accessed on 20/08/2023).

### Long-term hierarchical approach to classification

**Fig. 1:**
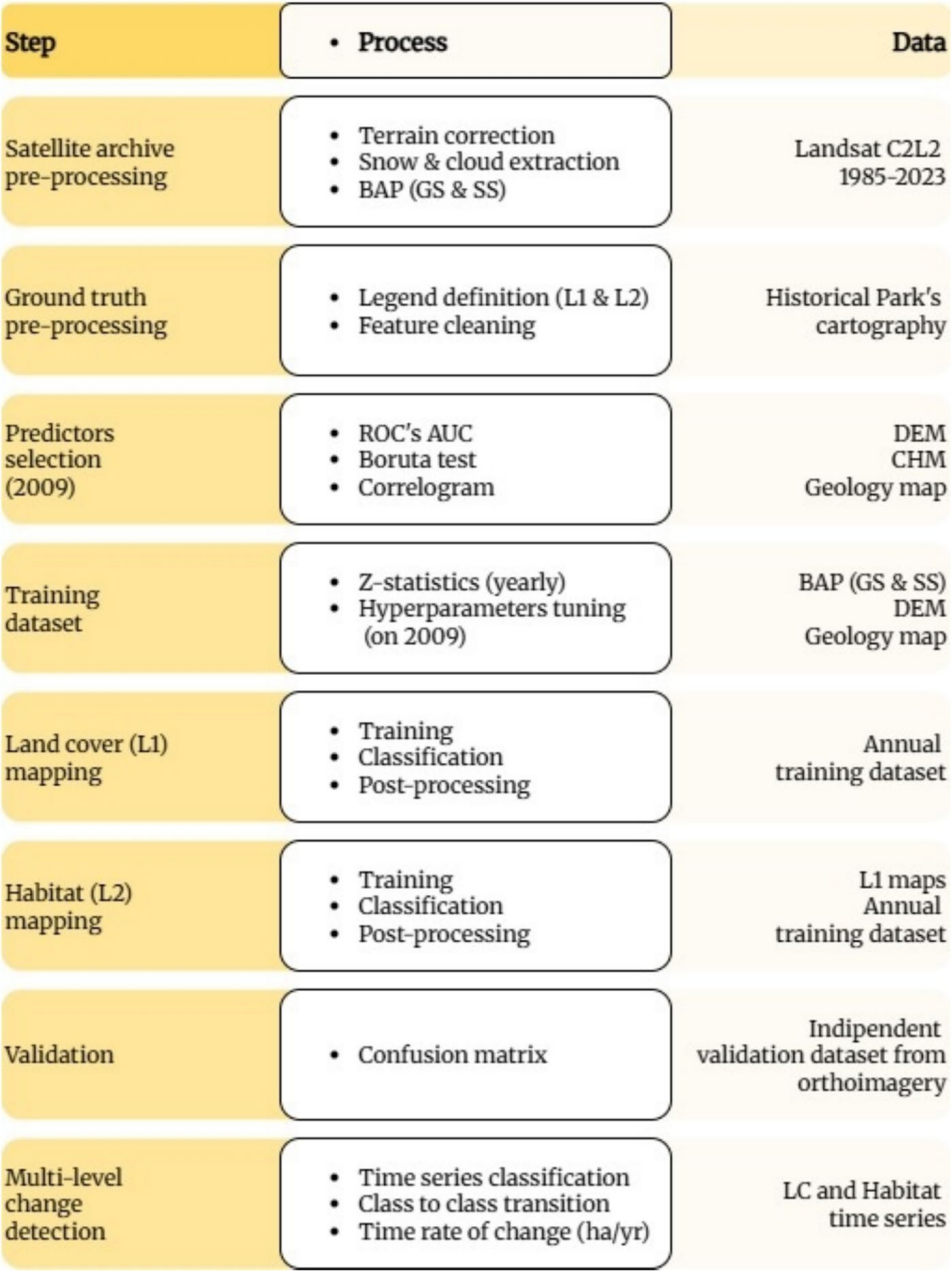
Flowchart simplified. The detailed flowchart in the Supplementary materials (Figure S2)

Unlike conventional classification approaches that directly assign habitat types (Agrillo et al., 2021; Szporak-Wasilewska et al., 2021), the proposed method follows a hierarchical approach: first land cover classification and then habitat mapping (Haest et al., 2017; Punalekar et al., 2024) (Fig. 1). This ensures that each habitat is classified only within its respective land cover target category, significantly reducing misclassification errors caused by spectral similarities between unrelated habitat types.

### Dataset and historical cartography

The training datasets were derived from the LULC cartography edited by the Park’s authority. This historical Park’s cartography was created during the years spanning from 2009 to 2011 and built based on an analysis of the vegetation from aerial photogrammetric surveys, ancillary cartography, and the field knowledge of the territory’s vegetation, ensuring excellent reliability. To adapt the cartography for training on Landsat imagery (30 m), several cleaning steps were implemented. This process involved removing patches smaller than the minimum mapping unit (MMU) selected, equal to a single pixel (i.e., < 900 m2), and ensuring that no patch dimension was smaller than 30 m. Additionally, we adjusted the legend to align with the semantic content appropriate for Landsat. The final legend adopted consists of 8 land cover types, shown in Table 1. The original legend and the translation in the adopted legend are reported in Table S3.

**Table 1:** Adopted legend: land cover types (Level 1, L1) (Chytrý et al., 2020; Kosztra György Büttner & Hazeu Stephan Arnold, 2019) and habitats (Level 2, L2) for target vegetation classes. Some habitats are identified by the Natura 2000 code reported in EU Habitats Directive, Annex I (Council Directive 92/43/EEC of 21 May 1992). A distinction is made between priority or particularly endangered habitat types which are marked with an asterisk (*)

L1	Land cover	Description	L2	Habitat
1	Rocks	These types are characterized by a low and sparse vegetation cover, specialized of these extreme habitats	-	-
2	Snow and glaciers	Perennial snows and glaciers	-	-
3	Water	Lotic and lentic waters	-	-
4	Broadleaved	These types are mainly located at lower elevations (up to 1500 m a.s.l.)	41	Scrubland and woods of Maple, linden and ash trees
			42	Oak forest
			43	Mixed broadleaved forests
			44	Mixed hygrophilous woods of broadleaved trees [incl. 91E0]
			45	Chestnut groves [incl. 9260]
			46	Beech forests [incl. 9110, 9130, 9150]
5	Coniferous	This type spread in the study area from 1500 to 2200 m a.s.l	51	Fir forests
			52	*Larix decidua* and/or *Pinus cembra* forests [9420]
			53	Mountain pine forests (*Pinus uncinata*) [9430]
			54	Mixed coniferous forests
			55	Sparse coniferous forests
			56	Spruce forests [9410]
			57	Scots pine forests
6	Grassland	Montane and subalpine grasslands are maintained by cattle grazing; in case of abandonment, encroachment causes a rapid vegetation dynamics to shrublands and then to forests. This evolution is present up to the actual limit of forests and above it in some valleys	61	Subalpine and alpine acidophilic grasslands [6150, 6230*, 36.33, 36.52]
			62	Subalpine and alpine calcicolous grasslands [6170, 36.12]
			63	Hydrophilous tall herb communities of the Alpine plain [cod. 6430 p.p.]
			64	Arid and thermophilic grasslands [incl. 6210, 6240*]
			65	Montane grasslands [incl. 6520]
7	Shrubland	Shrubs are an azonal vegetation type. Can be stable shrublands or involved in a succession stadium of grassland encroachment	71	Woody riparian vegetation of watercourses [incl. 3230, 3240]
			72	Green alder shrubs
			73	Shrubland (without distinction of species)
			74	Sub-arctic shrublands with *Salix* sp. [4080]
			75	Subalpine and alpine heaths [4060]
8	Wetland	Includes transitional peat bogs (code 7140), marshes with small basophilic sedges (code 7230), marches with small acidophilic sedges (code 54.4) and pioneer herbaceous vegetation of alpine watercourses and other herbaceous riparian vegetation (code 3220)	-	-

### Retrospective ground truthing using the Z-statistic

The Park’s historical cartography, following the cleaning steps previously described, was used to generate annual training datasets consisting of pure pixels for each land cover class. This was accomplished using the Z-statistic approach within a cross-correlation analysis (CCA) framework. In remote sensing, the Z-statistic is applied in CCA to detect land cover changes by comparing a reference land cover map at time T_1_ with a recent multispectral image at time T_2_. The method calculates, for each pixel, how much its spectral signature at T_2_ deviates from the expected mean signature of its class, as defined in T_1_ (Civco et al., 2002; Tarantino et al., 2016). In this study, the Z-statistic was computed across a stack of selected predictor variables, which varied from year to year, except for topographic and geological layers, which were assumed to remain stable over time.

The historical cartography was treated as the land cover reference map (T_1_), while the predictor variables (e.g., satellite-derived indices, CHM) were considered T_2_. The objective was to identify and select pixels lying near the center of the z-statistic distribution for each land cover class (see Equation S1), as these are most likely to represent “pure” class members (Tarantino et al., 2016). In contrast, pixels located in the distribution tails were assumed to have mixed or transitional characteristics and were excluded.

To construct the initial training dataset, the cleaned historical land cover map was intersected with the predictor layers for the year 2009, selected because it coincided with the publication year of most of the historical maps and included a canopy height model (CHM) not available in other years. From this intersection, the purest pixels were selected using the Z-statistic approach.

This step is repeated considering each year as T2, retaining only pixels that showed low deviation from the reference class signature (i.e., those located near the center of the z-statistic distribution). This ensured the exclusion of pixels potentially affected by land cover change, which are typically found in the tails of the distribution. In this way, the method systematically excludes changing or mixed pixels and selects only those that remained stable with high confidence. Hence, the Z-statistic method was applied to extract a reliable (i.e., unchanged and pure) subset of pixels by combining historical land cover data with satellite-derived predictors that are used as a training dataset to perform predictor selection via statistical tests and to train yearly random forest (RF) classifiers, subsequently employed to produce the corresponding land cover maps.

In summary, the Z-statistic allowed for the generation of retrospective ground truth datasets that served as the foundation for consistent and robust land cover mapping across multiple years.

### Predictors selection

The predictors were selected based on the method outlined by Desjardins et al. (2023). This approach integrates three complementary techniques: first, the receiver operating characteristic area under the curve (ROC AUC) (Kuhn & Johnson, 2013) was employed to assess the ability of each predictor in discriminating each class. We chose to discard the predictors with AUC < 0.75 in all the classes (Table S4); second, the Boruta test (Kursa & Rudnicki, 2010) was utilized to identify the optimal predictor combination (Figure S3); and third, correlation (Kendall, 1938) was analyzed to minimize multicollinearity (Figure S4). In total, 38 predictors were selected (Table S6).

### Random forest

The tuning of the hyperparameters was performed using the *tuneRanger* function of the *ranger* package (Wright & Ziegler, 2017), by setting 70 iters and a selection based on the best accuracy. The tuning with 100 trees led to the selection of optimal hyperparameters as follows: the number of variables randomly sampled at each split (*mtry*) was set to 16, the minimum size of terminal nodes (*min.node.size*) to 4, and the sample fraction to 0.51. Training and classification were performed using an ensemble approach of 5 RF models, trained separately for each year, aggregated into a final classification through majority voting. The training dataset dimensions are reported in Table S5. Variable importance plot is reported in Figure S5.

#### Post-processing

First, a reclassification based on the 39-years’ time series was performed based on the following rules: (i) pixels with missing values were filled by assigning the most frequent class within a temporal window of three preceding and three following years; (ii) pixels classified as “Water” (class 3) for more than half of the time series were reclassified as “Water” across all time layers to reflect persistent water bodies; (iii) pixels assigned to a unique class in a single year, with all other classifications consistently belonging to other classes, were reclassified to the most frequently assigned class across the time series. Secondly, a temporal aggregation of about each 10 consecutive years was conducted, in which each pixel is labelled as the most frequent value of that time period (1985–1995; 1995–2005; 2005–2015; 2015–2023). This series of post-processing steps helped to ensure consistency and reliability in the temporal classification data.

#### Validation

Ten independent ground truth datasets, generated manually through photointerpretation of available orthoimages (Table S7), served as the reference for constructing confusion matrices to assess the classification accuracy of the land cover maps (L1) of the corresponding years. The accuracy of the habitat map (L2) of the year 2009 was assessed by comparison against the original cartography of the Park’s authority. Overall metrics, i.e., overall accuracy (OA), balanced accuracy (BA), and Cohen’s kappa (K) coefficient, were calculated to evaluate the overall performance (Congalton & Green, 2019). Additionally, class-specific metrics, F1-score (F1), producer’s accuracy (PA), and user’s accuracy (UA), provided a detailed assessment of the performance for each land cover class (Foody, 2002).

Moreover, to assess the accuracy and reliability of the hierarchical classification and retrospective habitat mapping approach, we employed complementary validation metrics: the true skill statistic (TSS) (Allouche et al., 2006) and weighted accuracy (WA) (Geetha et al., 2024). These metrics are particularly relevant in habitat mapping, where rare habitats might otherwise be underrepresented in standard accuracy assessments.

#### Change detection

**Fig. 2:**
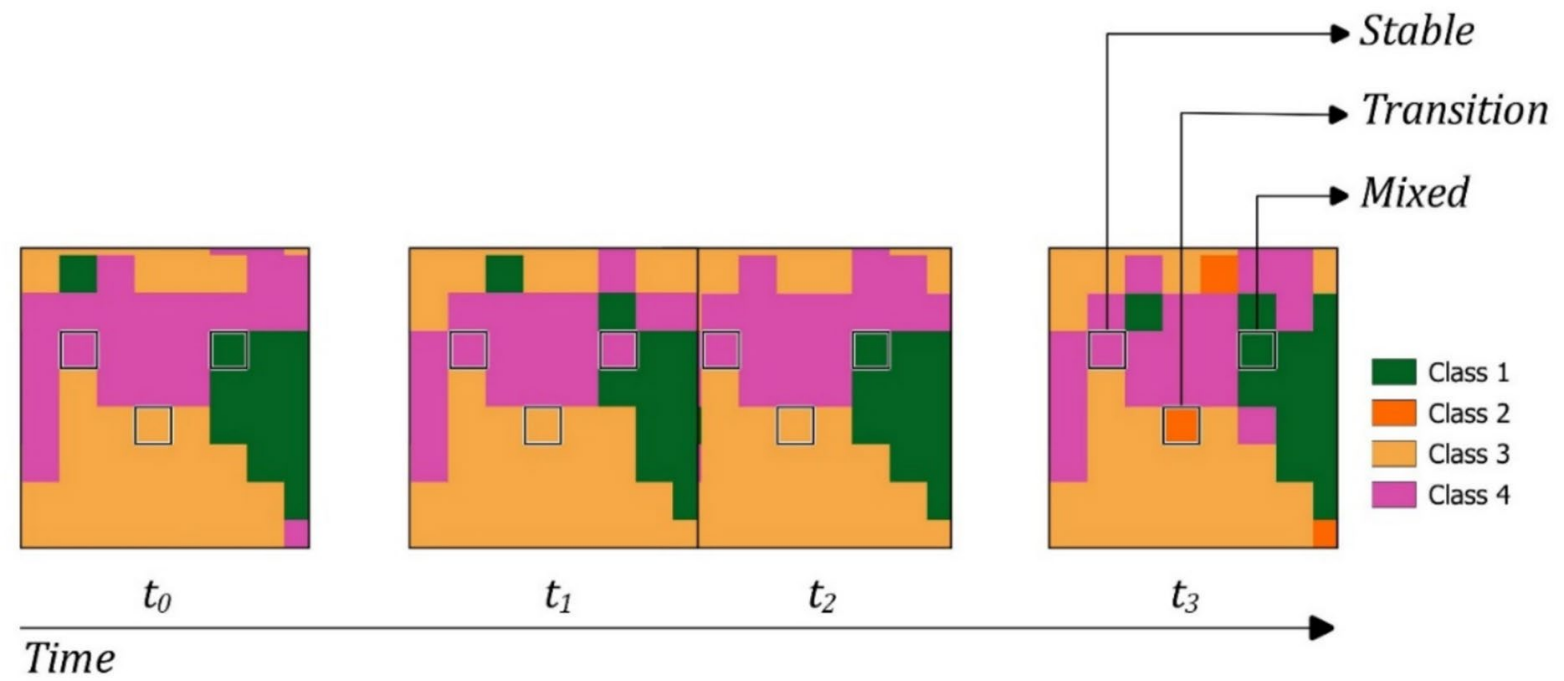
Pixel classification based on the time series, in this case the five 10-year temporal aggregation maps (t_0_ = 1985–1995; t_1_ = 1995–2005; t_2_ = 2005–2015; t_3_ = 2015–2023)

Each pixel was classified based on its change pattern over the study period. Three categories were defined: (1) *pure stable pixels*, which maintained a single, consistent land cover class across the entire time series, indicating no change; (2) *mixed pixels*, characterized by irregular changes between two or more classes without a consistent trend, suggesting a heterogeneous pattern; and (3) *transition pixels*, which displayed a clear, unidirectional shift from one class to another, signifying a stable transition in land cover. This classification framework facilitated a comprehensive understanding of spatial dynamics by distinguishing between static, fluctuating, and change patterns over time (Fig. 2).

### Climate and greening trends

Monthly climate data (climate moisture index, potential evapotranspiration, precipitation amount, mean air temperature, and vapor pressure deficit) were retrieved from the global CHELSA v2.1 dataset (Karger et al., 2017), consisting of downscaled model output at a 30 arc-sec resolution. These data were used to investigate climatic trends over the time period under analysis. Analogously, to assess greening trends, the normalized difference vegetation index (NDVI) calculated directly from the yearly GS BAP composite images was employed.

## Results and discussion

**Fig. 3:**
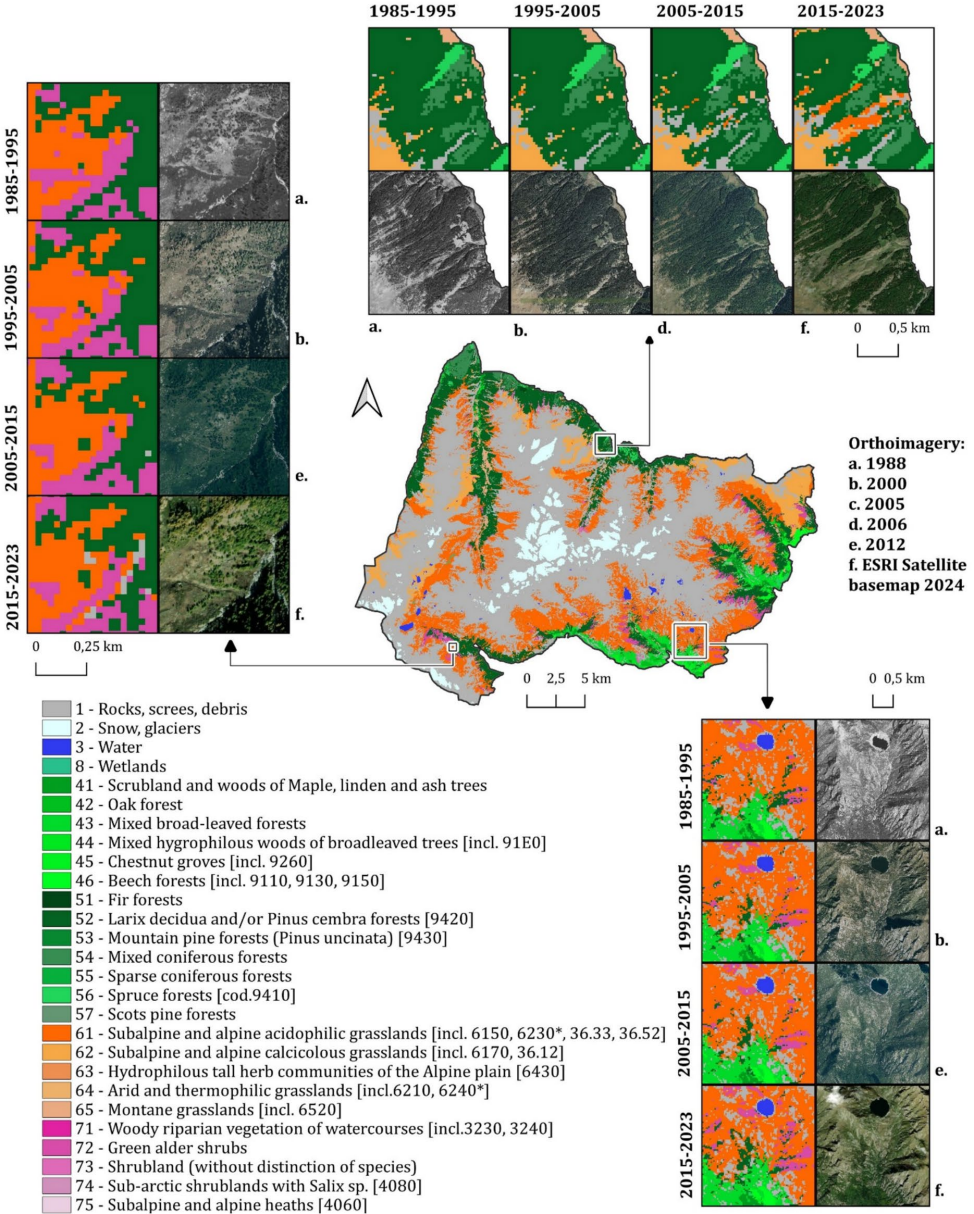
Long-term habitat mapping of the study area

For each year of the 1985–2023 period, first of all, a land cover map was generated and then habitat maps, resulting in a 39-year time series (Fig. 3). The choice of using the Park’s cartography to build the training dataset aimed to create a largely automated, replicable workflow (Sittaro et al., 2022). Many parks have high-quality mapping produced by experienced operators through photointerpretation and field surveys, as required by the Council of the European Union, (1992) directive. However, such manual mapping is time-consuming, costly, and difficult to repeat, making it unfeasible to update the cartography and effectively monitor habitats on a large scale (Martínez-López et al., 2021). By leveraging existing cartography for algorithm training, classifications can be automated, facilitating protected area monitoring and providing an effective tool for protected area management. While relying solely on the Landsat archive necessitated some loss of spatial and spectral detail due to its moderate resolution, integrating additional earth observation (EO) data with higher spatial and spectral resolution could help preserve the geometrical and semantic richness of the original historical cartography. This enhancement would make the proposed method more scalable and adaptable, enabling more detailed and accurate reconstructions of past landscapes. In addition, the proposed approach allows the generation of a multi-year time series from a single map source, improving the temporal analysis and tracking of habitat and land cover changes, while also enabling the construction of models for predicting changes under different scenarios (Lafitte et al., 2024).

### Validation

**Fig. 4:**
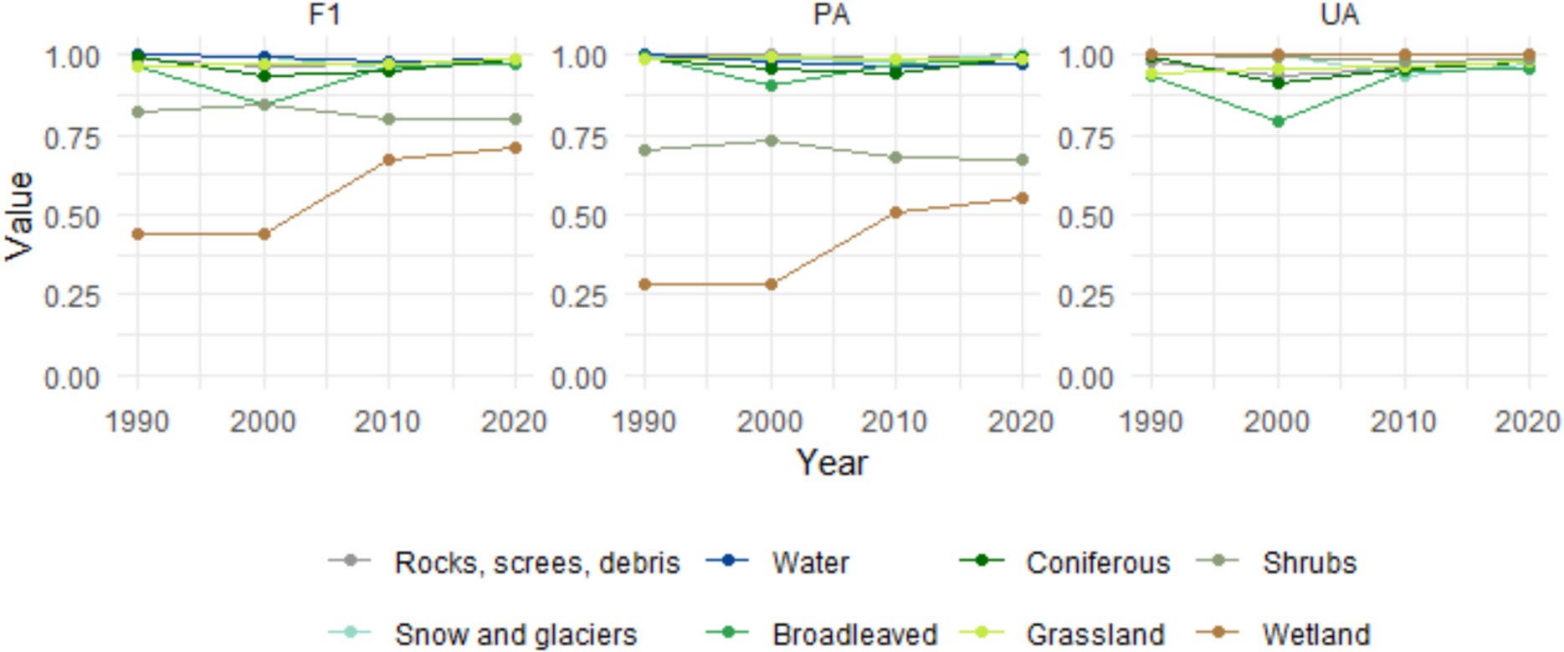
Class metrics for final, aggregated years, maps. F1, F-1 score; PA, producer’s accuracy; UA, user’s accuracy

Overall, the land cover classifier achieved excellent results. As shown in Table S8, accuracy remained highly consistent across most years, with metric values generally exceeding 0.96. Notable deviations are observed around the years 2000 and 2010. A visual inspection of the BAP images for these years revealed lower data quality, i.e., a low number of available images and/or radiometric low quality due to the different sensors onboard the Landsat constellation, e.g., Landsat 7 after SCL failure, which likely contributed to these dips. Despite these fluctuations, the metric values underscore the robustness of the classification approach over time. Figure 4 illustrates the temporal trends of class metrics. Many classes demonstrate consistent and high accuracy across all years, particularly for rocks, screes, and debris, snow and glaciers, and water, which maintain F1-scores, PA, and UA values near or above 0.90. Broadleaved and coniferous forests also exhibit stable and reliable accuracy metrics, with minor fluctuations over time. The shrubland class displays moderate PA values, indicating underestimation issues. In contrast, the wetland class shows significant variability in accuracy metrics, with pronounced dips in all metrics at several time points, indicating inconsistent model performance for this category. Visual inspection of the results confirms that the presence of wetlands was correctly identified; however, delineating their spatial extent proved unreliable. This limitation is likely influenced by seasonal variations in hydrological and vegetative conditions, which can significantly affect the detectability and apparent boundaries of wetland areas in remotely sensed imagery (Le Dez et al., 2021). Thus, this class necessitates the adoption of tailored mapping methodologies. Furthermore, wetlands occurred in small patches and should be investigated with VHR images. Due to its unreliability, the wetland class was excluded from further analysis to ensure the robustness of subsequent interpretations and conclusions. Class metrics before the post-processing step are shown in Figure S6.

**Fig. 5:**
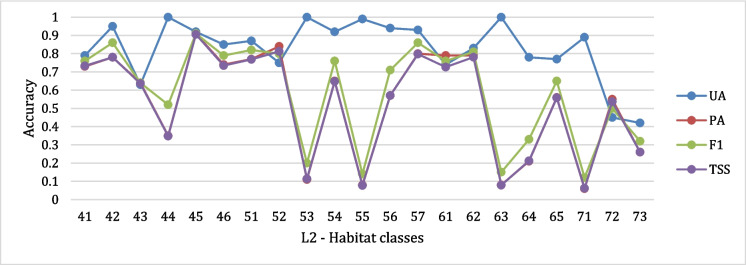
*Aggregation 2005*–*2015 habitats class metrics (L2). F1*, *F-1 score; PA*, p*roducer’s* a*ccuracy; UA*, u*ser’s* a*ccuracy; TSS*, t*rue* s*kills* s*tatistics. X-axis labels: L2 –* h*abitat classes: 41-Scrubland and woods of Maple, linden and ash trees, 42-Oak forest, 43-Mixed broad-leaved forests, 44-Mixed hygrophilous woods of broadleaved trees [91E0], 45-Chestnut groves [9260], 46-Beech forests [9110, 9130, 9150], 51-Fir forests, 52-*Larix decidua *and/or Pinus cembra forests [9420], 53-Mountain pine forests (*Pinus uncinata) [9430], 54-Mixed coniferous forests, 55-Sparse coniferous forests, 56-Spruce forests [9410], 57-Scots pine forests, 61-Subalpine and alpine acidophilic grasslands [6150, 6230*, 36.33, 36.52], 62-Subalpine and alpine calcicolous grasslands [6170, 36.12], 63-Hydrophilous tall herb communities of the Alpine plain [6430], 64-Arid and thermophilic grasslands [6210, 6240*], 65-Montane grasslands [6520], 71-Woody riparian vegetation of watercourses [3230, 3240], 72-Green alder shrubs, 73-Shrubland (without distinction of species)

Overall metrics of the 2005–2015 aggregated habitat map are WA = 0.83, OA = 0.84, and K = 0.77, showing an overall good performance even though not excellent. Classification performance, assessed through class metrics (UA, PA, F1, and TSS), varied considerably across habitat types (Fig. 5). Classes with higher accuracy generally corresponded to habitats characterized by large, spatially cohesive patches (e.g., *L. decidua* and/or *Pinus cembra* forests, mixed broadleaved forests). Conversely, classes exhibiting lower accuracy (e.g., green alder shrubs, woody-riparian vegetation, montane grasslands) were typically associated with small, fragmented patches or transitional ecotones, which are more prone to spectral confusion and misclassification. Low PA values indicate an underestimation of habitat classes, suggesting that a significant number of reference samples were not correctly classified. However, the high UA values suggest that the labels assigned in the classification are generally reliable, with a low rate of commission errors. In other words, while some habitats are underrepresented in the map (low PA), those that are labelled as a given class are likely to be correct (high UA). We further investigated how spatial characteristics of habitats influenced classification accuracy by correlating per-class accuracy scores with landscape metrics derived using LecoS (Jung, 2016). Results show that mean patch area was moderately correlated with PA (*r* = 0.60), F1-score (*r* = 0.59), and TSS (*r* = 0.58) (Figure S7), indicating that habitats with more extensive and internally homogeneous patches are classified more accurately. Edge length and shape complexity also showed similar positive associations. Conversely, UA appeared largely independent of spatial structure, likely reflecting confusion with spectrally similar classes. This suggests that pixel size is a limitation for accurately detecting ecotones, which are areas most active and affected by climate change (Smith & Goetz, 2021).

### Multi-decades of vegetation and habitats dynamics

**Fig. 6:**
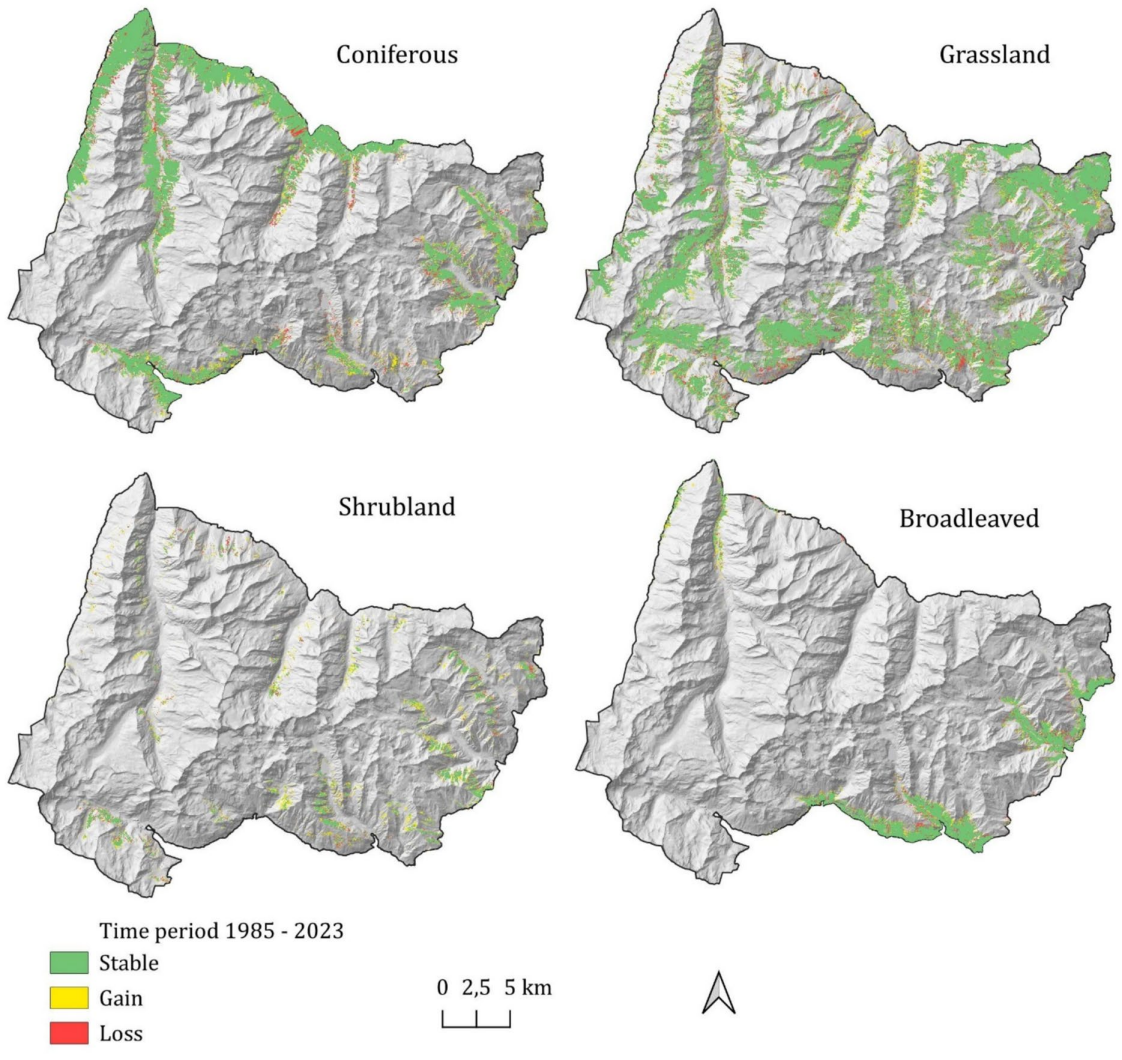
Vegetation dynamics

Vegetation dynamics are shown in Fig. 6, which illustrates the area of expansion, contraction, and stability of the different vegetation land cover classes. Areas subject to dynamism, loss or gain, are concentrated at patch boundaries, while the cores show steadiness. The temporal trends, expressed as rates of change per area (ha year^−1^), reveal distinct patterns of expansion and contraction among the different habitats across the two regions and across four altitudinal bands (Figure S8): mountain (1400–1900 m), sub-alpine (1900–2300 m), alpine (2300–2700 m), and nival belt (2700–4000 m). Grasslands experienced the most dramatic contraction, shrinking by 10.09 ha year^−1^. Changes have occurred mainly in the Piedmont side of the park (–8.27 ha year^–1^) and in the subalpine belt (−6.22 ha year^−1^). The observed trend resonates with the broader literature reporting that subalpine and alpine grasslands are particularly sensitive to climate change and land-use abandonment, leading to shrub encroachment (Cannone et al., 2022; Pittarello et al., 2016). Subalpine and alpine acidic grasslands [6150, 6230*] represented the largest extent of loss (about 200 ha), while in percentage the bigger contraction regards Montane grasslands [6520] (−5.4%). Similarly, subalpine and alpine calcicolous grasslands [6170, 36.12] show a moderate contraction (−2%). The only grassland habitat expanding is the arid and thermophilic grasslands [6210, 6240*] (+12.1%). Broadleaved forests remained relatively stable, displaying an average annual loss of 0.71 ha year^−1^, while coniferous forests declined more markedly, losing 4.41 ha year^−1^, occurring in the Aosta Valley region. The relatively high stability of broadleaved and coniferous forests in the study area is in partial contrast to findings in other Alpine regions in the European Alps, where an increase in forest stands has been documented as a result of land-use abandonment and climate warming (Anselmetto et al., 2024; Garbarino et al., 2013; Gehrig-Fasel et al., 2007; Seidl et al., 2011) probably due to the fact that the period analyzed is limited to the latter part (1985–2023) of the postwar period and the approach used is poorly sensitive to changes at the level of ecotones. Shrublands demonstrated the fastest growth of all classes, gaining 10.01 ha year^−1^, indicating a robust encroachment into former grassland areas and a possible recovery of previously grazed sites, equally on both sides of the park, mainly in the subalpine belt (+7.15 ha year^−1^). Similar patterns of shrub expansion at the expense of open grasslands have been documented across the European Alps and other mountainous regions worldwide (Khazieva et al., 2022; Soubry et al., 2022). In the alpine belt, shrubland expansion persisted, with an annual gain of 1.24 ha year^−1^, reflecting the progressive establishment of woody vegetation in the upper alpine zone. Similar upward shifts of shrubs and treelines have been documented throughout the European Alps and other mountain ranges, reflecting biotic responses to warming temperatures, altered precipitation regimes, and also abandonment of grazing activities (Harsch and Bader, 2011; Harsch et al., 2009; Rumpf et al., 2019). The relatively stable or moderately declining patterns in forest cover at upper elevations align with treeline dynamics observed in other studies, where forest lines may lag climatic shifts due to disturbance regimes or soil development limitations (Gehrig-Fasel et al., 2007).

**Fig. 7:**
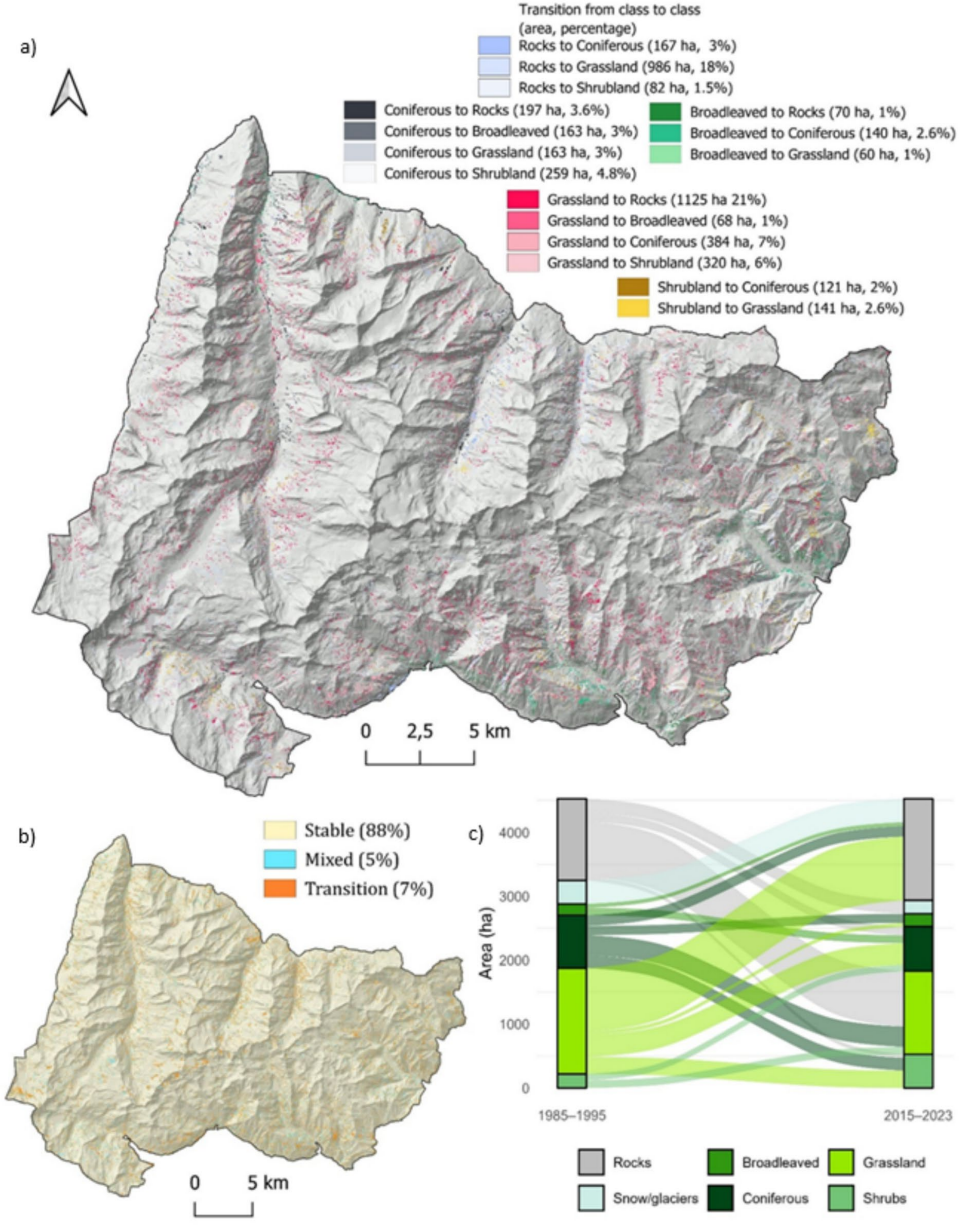
*Land cover transitions from 1985 to 2023. ****a**** Map of transitions; ****b*** m*ap of stable–mixed and transition areas; ****c*** a*lluvial map. Transitions lower than 1% were excluded. Standalone full-page maps of ****a**** and ****b**** are available in the SM *(*respectively *Figures S10 and S11)

**Fig. 8:**
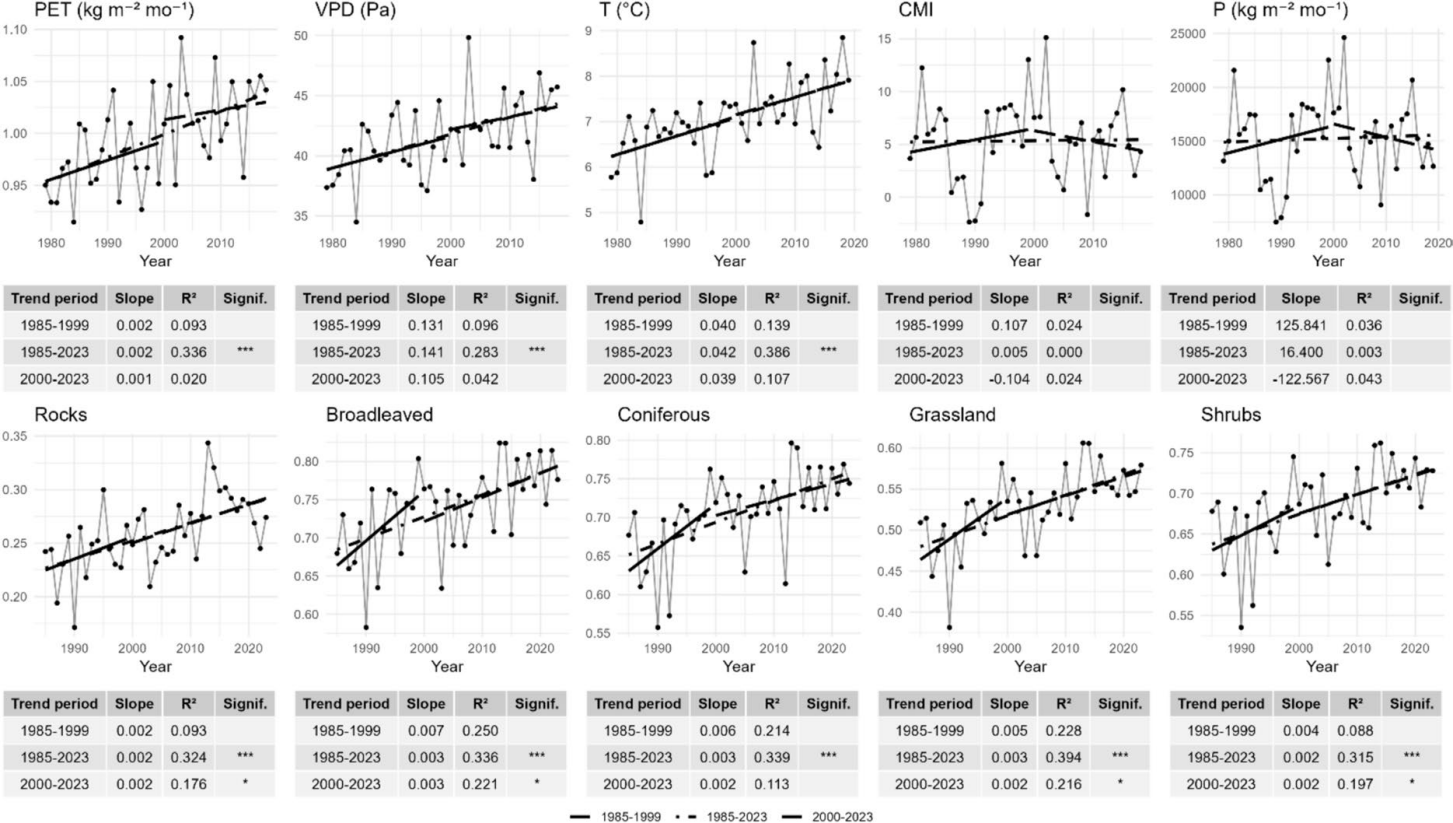
Upper row: climatic variables (PET, potential evapotranspiration; VPD, vapor pressure deficit; T, temperature; CMI, climate moisture index; P, precipitation), CHELSA v2.1 (Karger et al., 2017). Lower row: NDVI trends per class. Significance stars: *p < 0.05, **p < 0.01, ***p < 0.001. Both trends are calculated for the vegetative season (from May to September)

The class-to-class transitions and pixel types are illustrated in Fig. 7, with pixels classified as stable, transitional, or mixed. Stable pixels, representing consistent land cover types over time, i.e., the total or the greater part of the pixel covered by a unique land cover class, dominate the landscape, covering approximately 88% of the area (~ 62,000 ha). To ensure a robust interpretation of land cover dynamics and minimize the influence of classification noise or artefacts, the class-to-class changes analysis focused exclusively on pixels exhibiting actual transitions between classes over time. Transition pixels, which reflect the stable transition from one class to another, account for 7% (~ 5000 ha) and are indicative of ongoing vegetation changes, comparable to patterns described in other European Alpine systems (Carlson et al., 2017a, 2017b; Filippa et al., 2019a, 2019b; Lamprecht et al., 2018; Walther et al., 2005). The dominant transition, i.e., grassland to rocks (21%, 1125 ha), could reflect a decline in vegetation cover in marginal and higher areas and effects of drought due to the altered temperatures and precipitation regime (Fig. 8) or soil erosion. This could align with the observed “browning” phenomenon described at the landscape level in the last years in other sites (Liu et al., 2021, 2023; Treharne et al., 2019). In contrast, the rocks to grassland transition (18%, 986 ha) indicates an expansion of the vegetation on rocky substrates. The NDVI trends (Fig. 8) mirror a broader “greening” trend reported in alpine environments (Choler et al., 2021; Rumpf et al., 2022). These transitions of almost equal magnitude (21% and 18%), affecting a very small area (~ 1000 ha), together with the increasing NDVI trends (Figure S9), even from a visual analysis, where these pixels appear scattered and heterogeneous, suggest misclassifications rather than true grasslands to rocks transitions. These classification errors likely stem from the complex topography of mountainous areas, where radiometric distortions and shadows can be mitigated through corrections but cannot be fully eliminated. The grassland to conifer (7%, 384 ha) and grassland to shrubland (6%) transitions show a process of encroachment of subalpine grasslands, which reflects ongoing ecological transformations driven by climate change, as well as the loss of traditional agricultural and grazing practices in mountainous regions (MacDonald et al., 2000; Tasser & Tappeiner, 2002). This is also witnessed by the regional analysis, which shows that the pasture loss is occurring mainly on the Piedmont region’s side of the park, more subject to land abandonment. Conifer to shrubland (4.8%) transitions indicate phenomena of forest degradation or reverse succession, potentially caused by disturbing events (avalanches, wind, pathogens, fire) and increasing water stress, consistent with the recent reduction in CMI and precipitation (Fig. 8), as well as possible ecotonal dynamics in marginal environments (Holtmeier & Broll, 2005; Temperli et al., 2012). The varied responses among classes demonstrate the intricate interplay between land abandonment, climate change, and ecological succession. Similar gradients have been observed in other protected alpine areas, where reduced grazing pressures and warming temperatures collectively drive heterogeneous and often non-linear vegetation responses (Boulangeat et al., 2014; Kidane et al., 2012; Pauli et al., 2012; Ramskogler et al., 2025). It is interesting also to note that the greening trend is “broken” into two periods that give rise to two patterns with two different slopes (steeper in the first and lower in the second), particularly pronounced for broadleaves, conifers, and grasslands (Fig. 8). Mixed pixels, representing areas that sway from one class to another, account for 5% (~ 3500 ha) of the study area. They mirror the complexity noted in previous studies of mountainous landscapes (Dufour et al., 2006; Orlandi et al., 2016), where microtopographic variations, local disturbance regimes, and heterogeneous land-use histories contribute to a mosaic of multiple coexisting plant communities within a relatively small area.

### Strengths and limitations of the proposed workflow

The suite of land cover and habitat mapping methodologies has evolved markedly over the past two decades. Early efforts relied on simple, bi‐temporal change detection of satellite scenes (Maggi et al., 2004). More recent work, which relies on multi-temporal satellite images and machine‐learning classifiers, has reached excellent results (Hohensinner et al., 2021; Kindermann et al., 2023). Nevertheless, habitat mapping remains a challenging task, particularly for retrospective studies, due to the amount of training data required (Mücher et al., 2023).

The proposed framework presents several advantages over traditional habitat mapping methodologies. First, the hierarchical structure significantly reduces classification errors by restricting habitat classification to ecologically plausible land cover categories. Secondly, the Z-statistic provides a robust measure for pure pixel selection by quantifying deviations from historical distributions, allowing us to reconstruct past habitat states without requiring continuous multi-temporal in situ data (Le Dez et al., 2021). Third, the proposed method can be applied to different landscapes and pre-existing cartographies, offering flexibility in habitat monitoring applications for large-scale implementation without requiring extensive ground-truthing efforts (Le Dez et al., 2021). This is enabled by the reliance on open-access data, including spectral and phenological information from multispectral satellite sources, such as the Landsat archive, and topographic data derived from DEM. An additional strength of the proposed method lies in its independence from ancillary variables such as soil data, which, although highly predictive of habitat distribution, can introduce operator-related biases and inconsistencies when applied at broader scales. Highlighting this independence further strengthens the case for adopting the approach at supranational scales, as illustrated by Bourdouxhe et al. (2023), who emphasize the challenges posed by such biases in vegetation modelling efforts. Compared to many standard RF applications, the method improves classification robustness by employing multiple ensemble models, which reduce the risk of overfitting and increase classification stability. It also enhances the final outputs by applying a temporal post-processing filter that eliminates noise and outliers. Finally, the use of seasonally derived variables, such as spectral indices for growing/senescence season, represents a relatively unexplored yet promising approach in alpine habitat mapping, where phenological shifts due to altitude are particularly pronounced (Cornelius et al., 2013; Inouye & Wielgolaski, 2024). To address this issue, we introduced specific criteria in the pixel selection during BAP composing.

Despite its advantages, the proposed workflow has inherent limitations that should be acknowledged. A primary concern is the dependence on the quality of the historical input data (Mücher et al., 2023). Since the Z-statistic approach relies on deviations from an initial reference dataset, inaccuracies in the historical cartography would propagate, affecting the reliability of the reconstructed habitat maps. Another challenge involves the mapping of habitats under the MMU allowed by the spatial resolution of the satellite sensor (Mücher et al., 2023). Certain small-scale or patchy habitats may be underrepresented or misclassified due to resolution constraints, requiring additional validation through in situ data collection. This limitation could also affect the detection of early successional stages or small-scale disturbances, which are critical for conservation planning and ecosystem management. This is also reflected in mixed, heterogeneous pixels, which present a significant challenge in land cover classification and remain an open challenge in this study. Similar landscape complexity was reported by Rumpf et al. (2019) in alpine ecosystems, where shifts in vegetation composition often occur gradually rather than as abrupt class transitions. Detecting transitions between land cover classes implies the existence of a colonization rate, where gradual changes in fractional cover contribute to shifts from one class to another. This underscores the need for finer-scale approaches, including spectral unmixing, very high-resolution imagery (VHR), and ground surveys, to disentangle mixed signals and improve ecological interpretation (Gudex-Cross et al., 2017; Marsoner et al., 2023; Soubry & Guo, 2021; Xing et al., 2023). To enhance the applicability and precision of this methodology, several improvements can be explored. First, integrating multi-source remote sensing data, such as LiDAR and SAR, could provide additional structural and moisture-related information, improving habitat differentiation (Sittaro et al., 2022). Second, incorporating other machine and deep learning techniques, such as neural networks, could enhance the classification performance by learning complex spatio-temporal patterns between the predictive variables and the habitats’ distribution (Mücher et al., 2023). Automating the workflow, using cloud-based platforms, would enable near real-time monitoring of habitat and, thus, support protected area management (Martínez-López et al., 2021). Finally, while the method has demonstrated promising results in an alpine protected area, further testing is necessary in diverse biogeographical contexts to ensure its robustness and generalizability. Moreover, expanding the methodology to include per-pixel uncertainty assessments would improve the confidence in historical habitat reconstructions, allowing for more informed conservation decisions.

A critical aspect of advancing remote sensing and AI-based methods in ecology lies in their applicability beyond academic contexts. The proposed framework is designed to be both scalable and transferable, relying on open-access datasets and standardized procedures. By integrating long-term EO data with historical cartography, it highlights the value of retrospective analysis in understanding ecological dynamics. This makes it suitable for integration into existing conservation monitoring systems, particularly in data-scarce or logistically challenging regions such as alpine environments. By explicitly addressing the practical utility of the method, this study underscores the role of EO in supporting adaptive management strategies and long-term habitat conservation.

## Conclusions

From this study emerges the high stability of most of the Gran Paradiso National Park’s territory, with 88% of the area remaining unchanged over time. Comparison with previous investigations (Carlson et al., 2017a, 2017b; Choler et al., 2021; Filippa et al., 2019a, 2019b) confirms that while the trend is a general greening, the specifics of land cover transitions are strongly modulated by regional factors. We observe a grassland decline, particularly in the Piedmont side. The transitions between grassland and shrubs/coniferous reflect ecological successional trends due to traditional agropastoral activities abandonment and climate change, witnessed also by greening and climate variable trends. The 7% of the area in transition represents a significant subset to be monitored, as it is potentially sensitive or in a state of irreversible change. Future research will aim to identify and quantify the key drivers of observed land cover changes, with a particular focus on disentangling the roles of anthropogenic activities and climatic factors in shaping these dynamics. Pixel size represents a limitation in classifying mixed, heterogeneous pixels. In consequence, the approach is particularly reliable for compact and large patches, while it has proven to have a low reliability for small, sparse patches, such as ecotones. Despite this limitation, the hierarchical classification approach, coupled with a Z-statistic-based retrospective analysis, represents a novel and promising method for long-term retrospective habitat monitoring, which overcomes the need for multi-temporal in situ data. The proposed method can be easily applied to other protected areas since it relies basically on the availability of a limited set of data, i.e., a pre-existing cartography, a DEM, and a multi-temporal EO dataset. The findings emphasize the importance of integrating multi-temporal imagery and in situ validation to better differentiate between stable mixed and true transitional pixels. Such refined methods will improve the interpretation of landscape-scale vegetation dynamics and, thus, support evidence-based conservation strategies and inform management practices tailored to the unique socio-ecological conditions of alpine protected areas. In conclusion, this study not only advances methodological approaches in habitat mapping but also provides a practical tool to support conservation efforts. By enabling large-scale, repeatable assessments of habitat surface change, the framework can assist decision-makers and land managers in prioritizing actions and monitoring ecological outcomes, thereby reinforcing the role of remote sensing and AI in applied conservation science.

## Supplementary Information

Below is the link to the electronic supplementary material.

ESM 1 (DOCX 4.18 MB)

## Data Availability

The codes and the outputs generated and analysed during the current study are available in the following GitHub repository: https://github.com/chiararik/GPNP-habitat-mapping.
