# Supplementary material for: Unravelling decades of habitat dynamics in protected areas: A hierarchical approach applied to the Gran Paradiso National Park (NW Italy)
Source: Environ Monit Assess. 2025 Oct 20;197(11):1216. doi: 10.1007/s10661-025-14669-0 (PMC12535940; doi:10.1007/s10661-025-14669-0)
Supplement: Supplementary file 1 — (DOCX 4.18 MB) [file 10661_2025_14669_MOESM1_ESM.docx]

**Supplementary Materials**


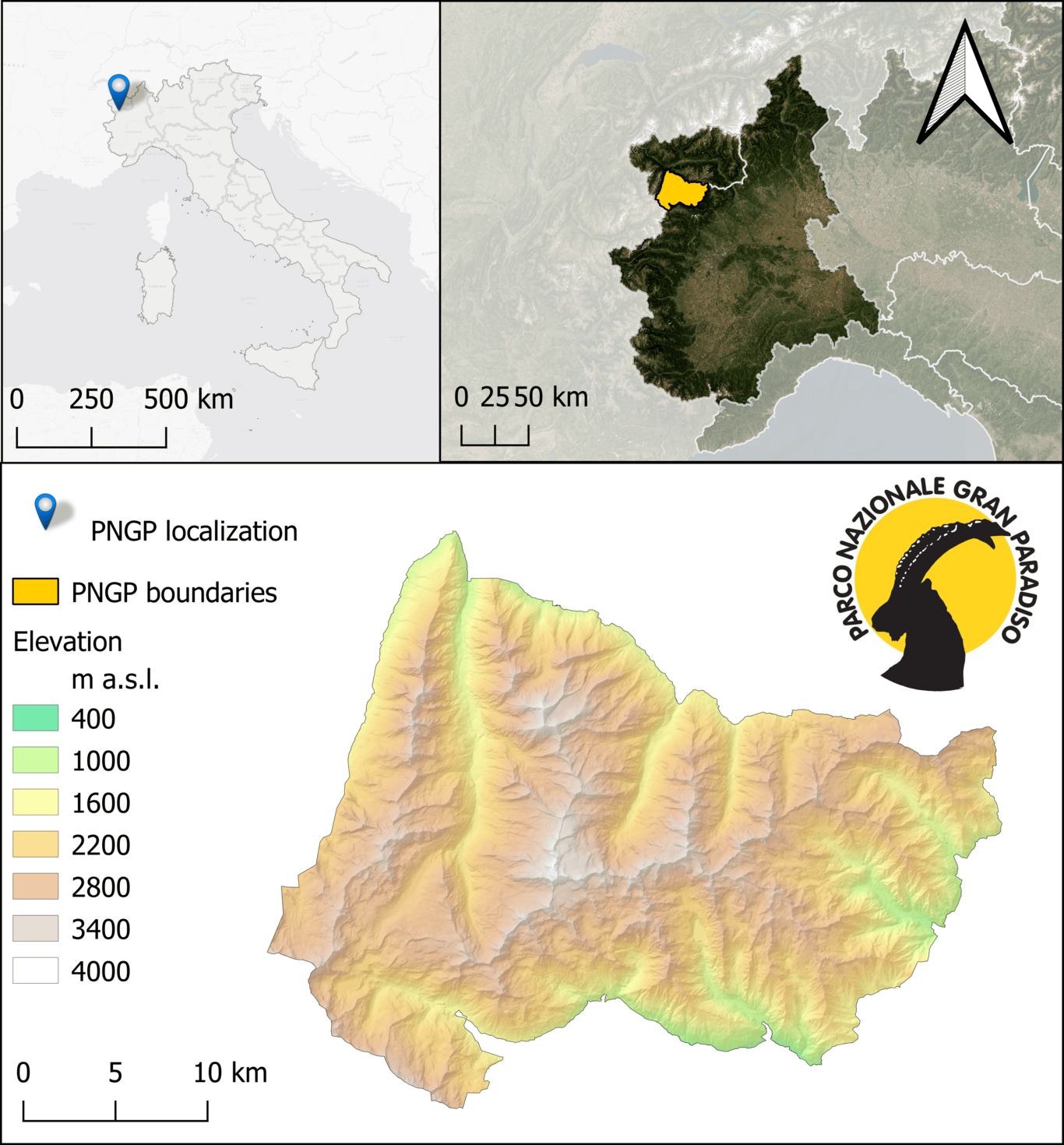


**Figure S 1** Study area: the Gran Paradiso National Park (GPNP), North-West Italy. For all maps CRS: EPSG 32632.

**Table S 1.** Parameters used for parametric scoring for BAP images compositing.

| **Parameter** | **Description** | **Weight** |
| --- | --- | --- |
| Distance to clouds and snow | It favours pixels distant from clouds, for better radiometric quality, and from snow, to select pixels further along in phenology after snowmelt during the growing season, and pixels not yet covered by snow during the senescence season. | 1 |
| Sensor | The aim is to avoid using Landsat 7 images collected after 31 May 2003, when the scan line corrector (SLC) failed. | 0.5 |
| Distance to central date | The aim is to try to use the pixels of the scenes acquired as close as possible to the same date central to the season (Days Of the Year 210 and 285, respectively for the growing and the senescence seasons), in order to have uniformity in the spectral response of the vegetation classes. | 0.25 |
| Haze-Optimized Transformation (HOT) | Has been developed to identify haze and thin clouds in Landsat imagery by Y. Zhang et al. (2002). For details, please refer to Zhu & Woodcock (2012) | 0.5 |
| Coverage | The aim is to favour images with more valid pixel coverage, i.e. higher radiometric quality. | 0.75 |
| Snow free area | Percentage of snow-free pixels. Newly introduced to favour the selection of image dates furthest from snow events during the senescence season and to favour pixels with the most advanced vegetative state post-snowmelt for the growing season. | 1 |

**Table S 2.** Full list of the predictors initially considered.

| **Spectral indices** | **Acronym** | **Formula** |
| --- | --- | --- |
| [Chlorophyll vegetation index](https://www.indexdatabase.de/db/i-single.php?id=391) | CVI | NIR*(red/((green)^2)) |
| [Coloration Index](https://www.indexdatabase.de/db/i-single.php?id=11) | CI | (red-blue)/red |
| [Enhanced Vegetation Index](https://www.indexdatabase.de/db/i-single.php?id=16) | EVI | 2.5*((NIR-red)/(NIR+6*red-7.5*blue+1)) |
| [Global Vegetation Moisture Index](https://www.indexdatabase.de/db/i-single.php?id=372) | GVMI | ((NIR+0.1)-(SWIR2+0.02))/((NIR+0.1)+(SWIR2+0.02)) |
| [Green leaf index](https://www.indexdatabase.de/db/i-single.php?id=375) | GLI | (2*green-red-blue)/(2*green+red+blue) |
| [Modified anthocyanin reflectance index](https://www.indexdatabase.de/db/i-single.php?id=215) | mARI | (green^(-1)-red^(-1))*NIR |
| [Modified Chlorophyll Absorption in Reflectance Index 1](https://www.indexdatabase.de/db/i-single.php?id=42) | MCARI1 | 1.2*(2.5*(NIR-red)-1.3*(NIR-green)) |
| [Modified Soil Adjusted Vegetation Index](https://www.indexdatabase.de/db/i-single.php?id=44) | MSAVI | (2*NIR+1-sqrt((2*NIR+1)^2-8*(NIR-red)))/2 |
| [Normalized Difference Vegetation Index](https://www.indexdatabase.de/db/i-single.php?id=59) | NDVI | (NIR-red)/(NIR+red) |
| [Normalized Difference Green NDVI](https://www.indexdatabase.de/db/i-single.php?id=401) | GNDVI | (NIR-green)/(NIR+green) |
| [Normalized Difference Burn Ratio](https://www.indexdatabase.de/db/i-single.php?id=53) | NBR | (NIR-SWIR1)/(NIR+SWIR1) |
| [Normalized Burn Ratio 2](https://www.usgs.gov/landsat-missions/landsat-normalized-burn-ratio-2) | NBR2 | (SWIR1-SWIR2) / (SWIR1 + SWIR2) |
| [Soil Adjusted Vegetation Index](https://www.indexdatabase.de/db/i-single.php?id=87) | SAVI | ((NIR-red)/(NIR+red+0.5))*(1+0.5) |
| Transformed Soil-Adjusted Vegetation Index | TSAVI | (0.7*(NIR-0.7*red-(-0.32)))/(-0.32*NIR+red-(-0.32)*0.7+1.5*(1+(0.7)^2)) |
| [Spectral Polygon Vegetation Index](https://www.indexdatabase.de/db/i-single.php?id=175) | SPVI | 0.4*(3.7*(NIR-red)-1.2*abs(green-red)) |
| [Tasselled Cap - brightness](https://www.indexdatabase.de/db/i-single.php?id=91) | SBI | 0.3037*blue+0.2793*green+0.4743*red+0.5585*NIR+0.5082*SWIR1+0.1863*SWIR2 |
| [Tasselled Cap - vegetation](https://www.indexdatabase.de/db/i-single.php?id=92) | GVI | -0.2848*blue-0.2435*green-0.5436*red+0.7243*NIR+0.0840*SWIR1-0.1800*SWIR2 |
| [Tasselled Cap - wetness](https://www.indexdatabase.de/db/i-single.php?id=93) | WET | 0.1509*blue+0.1973*green+0.3279*red+0.3406*NIR-0.7112*SWIR1-0.4572*SWIR2 |
| [Wide Dynamic Range Vegetation Index](https://www.indexdatabase.de/db/i-single.php?id=125) | WDRVI | (0.1*NIR-red)/(0.1*NIR+red) |
| [Simple Ratio Drought Index](https://www.indexdatabase.de/db/i-single.php?id=71) | RDI | SWIR2/NIR |
| [Normalized difference Built-up Index](https://doi.org/10.1117/12.2532322) | NDBI | (SWIR1 - NIR)/(SWIR1 + NIR) |
| [Moisture adjusted vegetation index](https://journals.plos.org/plosone/article?id=10.1371/journal.pone.0102560#pone.0102560-Jordan1) | MAVI | (NIR-red)/(NIR+red+SWIR) |
| [Atmospherically resistant vegetation index](https://www.sciencedirect.com/science/article/pii/S2590197420300148) | ARVI | (NIR-red-1*(red-blue))/(NIR+red-1*(red-blue)) |
| Normalized Difference Water Index | NDWI | (green - NIR) / (green + NIR) |
| [Bare Soil Index](https://www.mdpi.com/2073-445X/10/3/231) | BSI | ((SWIR2+red)-(NIR+blue))/((SWIR2+red)+(NIR+blue)) |
| [Normalized Difference Moisture Index](https://www.usgs.gov/landsat-missions/normalized-difference-moisture-index) | NDMI | (NIR - SWIR2) / (NIR + SWIR2) |
| Normalized Difference Snow Index | NDSI | (green-SWIR1) / (green+ SWIR1) |
| **Ancillary data** | **Acronym** | **Resolution (m)** |
| Geology | GEO | 1:100.000 |
| Canopy Height Model* (2008-2009) | CHM | 5 |
| **Topographic predictors** | **Acronym** | **Resolution (m)** |
| Elevation | DTM | 5 |
| Slope | slope | 5 |
| Aspect | aspect | 5 |
| Topography Roughness Index | TRI | 5 |
| Topographic Position Index | TPI | 5 |
| Diurnal Anisotropic Heat Index | DAHI | 5 |
| Curvature | crv | 5 |
| Topographic Wetness Index | TWI | 5 |

**Table S 3.** Original GPNP’s Authority LULC legend and the translation in the adopted legend. Classes that are numbered as 0 (in columns L1 or L2) have been excluded.

| **L1** | **Land cover** | **L2** | **Park’s LULC legend** |
| --- | --- | --- | --- |
| 0 | Bare soil | 0 | Disrupted environments |
|  |  | 0 | Quarry |
|  |  | 0 | Deposits of natural or artificial materials |
| 0 | Built-up | 0 | Bivouacs, mountain huts |
|  |  | 0 | Artifacts and infrastructures |
|  |  | 0 | Roads and trails |
|  |  | 0 | Structures and facilities for skiing |
|  |  | 0 | Buildings |
| 1 | Rocks, screes, debris | 0 | Calcareous and calc-scists screes |
|  |  | 0 | Calcareous and calc-scists cliffs |
|  |  | 0 | Siliceous screes |
|  |  | 0 | Siliceous cliffs |
| 2 | Snow and glaciers | 0 | Glaciers [8340] |
|  |  | 0 | Glaciers covered with debris [8340] |
|  |  | 0 | Perennial snows |
| 3 | Water | 0 | Lentic waters |
|  |  | 0 | Lotic waters |
|  |  | 0 | Water springs [incl. 7220*, 54.11] |
|  |  | 0 | Lentic waters with aquatic vegetation [incl. 3130] |
|  |  | 0 | Lentic waters partially buried |
| 4 | Broadleaved | 41 | Scrubland and woods of Maple, linden and ash trees |
|  |  | 42 | Oak forest |
|  |  | 43 | Mixed broad-leaved forests |
|  |  | 44 | Mixed hygrophilous woods of broadleaved trees [incl. 91E0] |
|  |  | 45 | Chestnut groves [incl. cod.9260] |
|  |  | 46 | Beech forests [incl. 9110, 9130, 9150] |
|  | - | 0 | Isolated trees or in small groups of different species (20-30%) |
|  | - | 0 | Mixed forests of conifers and broad-leaved trees |
|  | - | 0 | Reforestation |
|  | - | 0 | Cores of standing dead trees |
| 5 | Coniferous | 51 | Fir forests |
|  |  | 52 | *Larix decidua* and/or *Pinus cembra* forests [9420] |
|  |  | 53 | Mountain pine forests (*Pinus uncinata*) [9430] |
|  |  | 54 | Mixed coniferous forests |
|  |  | 55 | Sparse coniferous forests |
|  |  | 56 | Spruce forests [9410] |
|  |  | 57 | Scots pine forests |
| 6 | Grassland | 61 | Subalpine and alpine acidophilic grasslands [incl. 6150, 6230*, 36.33, 36.52] |
|  |  | 62 | Subalpine and alpine calcicolous grasslands [incl. 6170, 36.12] |
|  |  | 63 | Hydrophilous tall herb communities of the Alpine plain [6430 p.p.] |
|  |  | 64 | Arid and thermophilic grasslands [incl. 6210, 6240*] |
|  |  | 65 | Montane grasslands [incl. 6520] |
| 7 | Shrubs | 71 | Woody riparian vegetation of watercourses [incl. 3230, 3240] |
|  |  | 72 | Green alder shrubs |
|  |  | 73 | Shrubland (without distinction of species) |
|  |  | 74 | Sub-arctic shrublands with *Salix* sp. [4080] |
|  |  | 75 | Subalpine and alpine heaths [4060] |
| 8 | Wetland | 0 | Pioneer herbaceous vegetation of alpine watercourses [incl. 7240*] |
|  |  | 0 | Herbaceous riparian vegetation of watercourses [incl. 3220] |
|  |  | 0 | Marshes with small acidophilic sedges [54.4] |
|  |  | 0 | Marshes with small basophilic sedges [7230] |
|  |  | 0 | Transitional peat bogs [7140] |
| 0 | - | 0 | Parks, gardens, vegetable gardens, campsites, golf courses |
| 0 | - | 0 | Shadows |

**
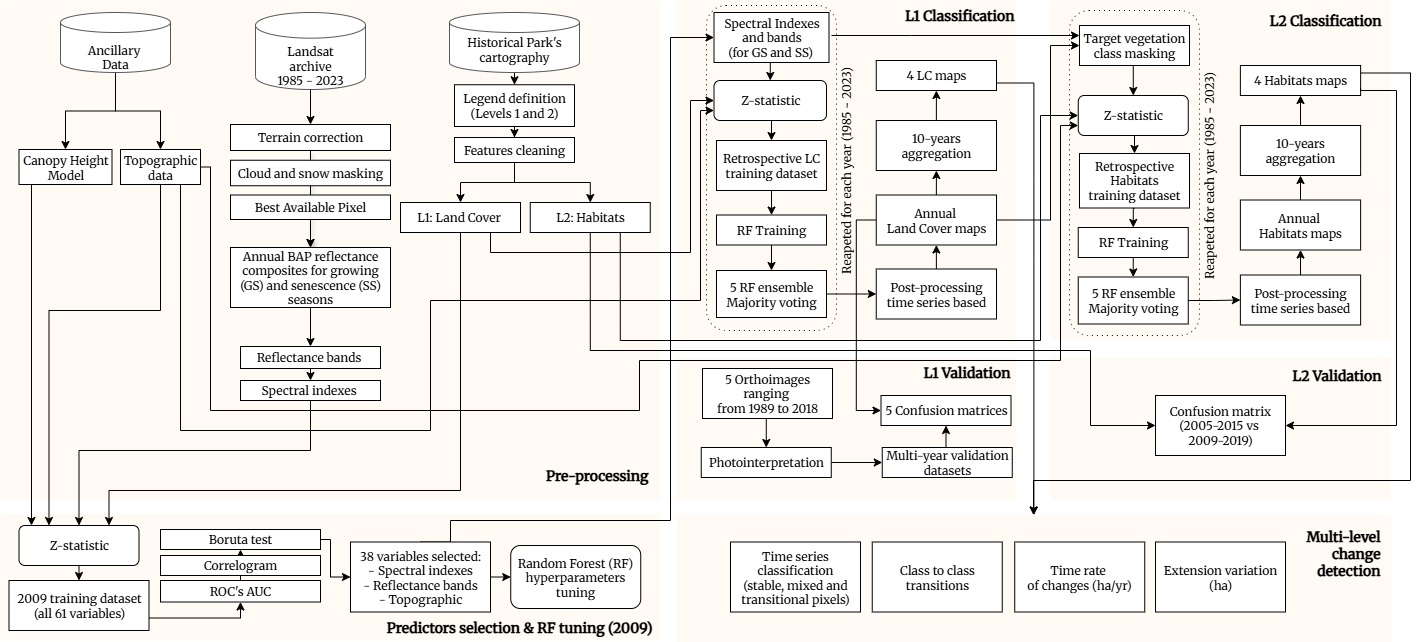
**

**Figure S 2.** Detailed flowchart of the proposed algorithm. ROC’s AUC = Receiver Operating Characteristic’s Area Under the Curve. L1 = Level 1 of the legend (Land cover classes); L2 = Level 2 of the legend (Habitats).

**Z-statistic formula**

The Z-statistic quantifies the multivariate distance between the pixel’s response and the expected response of the target class, as defined by Equation (S1):

$$Z_{jk}=\sqrt{\sum_{i=1}^{n} \left( \frac{r_{ijk}-\mu_{ic_{jk}}}{\sigma_{ic_{jk}}} \right)^{2}}$$

where:

*Z_jk_* is the Z-score for a pixel *jk* belonging of a given class

*i* is the predictor number

*n* is the number of predictors

*c_jk_* is the thematic class being considered,

*jk* is a pixel in the class

*r_ijk_* is the predictor value in *i* for pixel *jk*

*μ_icjk_* is the mean value in *i* of all pixels in each class *c_jk_*

*σ_icjk_* is the standard deviation of the predictor value in band *i* of all pixels in class *c_jk_*


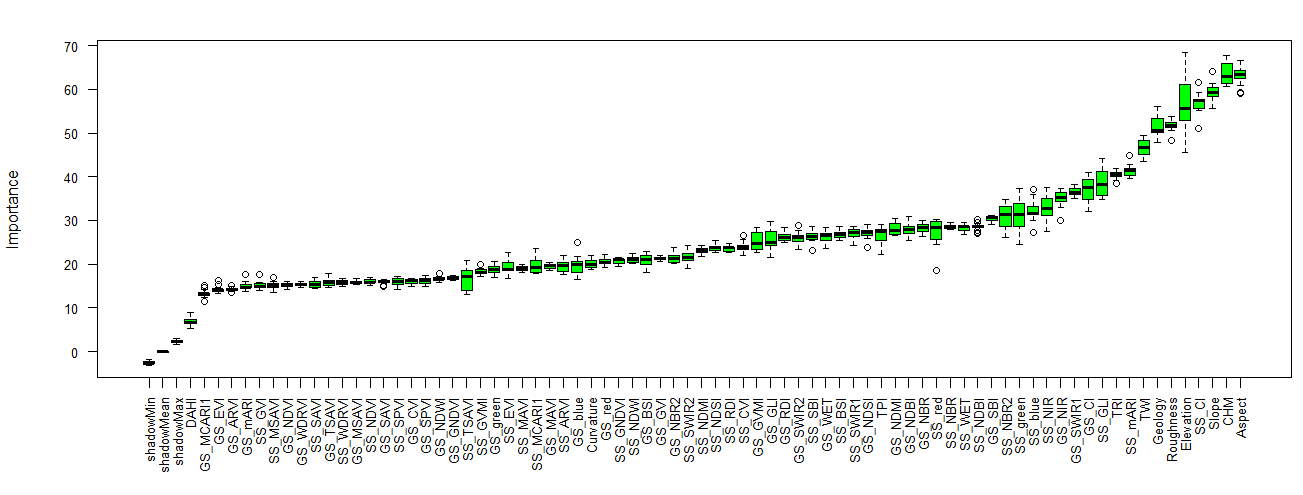


**Figure S3.** Predictors importance according to the Boruta test applied in 2009.

**Table S4.** Receiver operating characteristic’s area under the curve (ROC’s AUC).

| **Predictor** | **Rocks** | **Water** | **Snow** | **Broadleaved** | **Coniferous** | **Grassland** | **Shrubs** | **Wetland** |
| --- | --- | --- | --- | --- | --- | --- | --- | --- |
| GS_ARVI | 0.546 | 0.998 | 0.996 | 0.927 | 0.971 | 0.898 | 0.562 | 0.998 |
| GS_BSI | 0.977 | 0.996 | 0.991 | 0.977 | 0.977 | 0.977 | 0.977 | 0.996 |
| GS_CI | 0.939 | 0.939 | 0.939 | 0.939 | 0.939 | 0.939 | 0.939 | 0.701 |
| GS_CVI | 0.932 | 0.996 | 0.986 | 0.932 | 0.955 | 0.932 | 0.932 | 0.996 |
| GS_EVI | 0.853 | 0.990 | 0.970 | 0.928 | 0.971 | 0.876 | 0.853 | 0.990 |
| GS_GLI | 0.874 | 0.981 | 0.978 | 0.874 | 0.912 | 0.874 | 0.874 | 0.981 |
| GS_GNDVI | 0.960 | 0.998 | 0.995 | 0.960 | 0.963 | 0.960 | 0.960 | 0.998 |
| GS_GVI | 0.963 | 0.992 | 0.976 | 0.963 | 0.963 | 0.963 | 0.963 | 0.992 |
| GS_GVMI | 0.993 | 0.993 | 0.993 | 0.993 | 0.993 | 0.993 | 0.993 | 0.962 |
| GS_mARI | 0.672 | 0.989 | 0.976 | 0.745 | 0.938 | 0.715 | 0.672 | 0.989 |
| GS_MAVI | 0.962 | 0.999 | 0.996 | 0.962 | 0.969 | 0.962 | 0.962 | 0.999 |
| GS_MCARI1 | 0.937 | 0.988 | 0.963 | 0.937 | 0.968 | 0.937 | 0.937 | 0.988 |
| GS_MSAVI | 0.963 | 0.993 | 0.975 | 0.963 | 0.968 | 0.963 | 0.963 | 0.993 |
| GS_NBR | 0.993 | 0.993 | 0.993 | 0.993 | 0.993 | 0.993 | 0.993 | 0.889 |
| GS_NBR2 | 0.679 | 0.996 | 0.993 | 0.922 | 0.968 | 0.908 | 0.696 | 0.996 |
| GS_NDBI | 0.993 | 0.993 | 0.993 | 0.993 | 0.993 | 0.993 | 0.993 | 0.889 |
| GS_NDMI | 0.993 | 0.993 | 0.993 | 0.993 | 0.993 | 0.993 | 0.993 | 0.962 |
| GS_NDSI | 0.993 | 0.993 | 0.993 | 0.993 | 0.993 | 0.993 | 0.993 | 0.962 |
| GS_NDVI | 0.956 | 0.998 | 0.996 | 0.956 | 0.967 | 0.956 | 0.956 | 0.998 |
| GS_NDWI | 0.960 | 0.998 | 0.995 | 0.960 | 0.963 | 0.960 | 0.960 | 0.998 |
| GS_RDI | 0.993 | 0.993 | 0.993 | 0.993 | 0.993 | 0.993 | 0.993 | 0.962 |
| GS_SAVI | 0.961 | 0.994 | 0.978 | 0.961 | 0.968 | 0.961 | 0.961 | 0.994 |
| GS_SBI | 0.868 | 0.868 | 0.927 | 0.868 | 0.868 | 0.868 | 0.909 | 0.854 |
| GS_SPVI | 0.961 | 0.986 | 0.961 | 0.961 | 0.967 | 0.961 | 0.961 | 0.986 |
| GS_TSAVI | 0.725 | 0.983 | 0.945 | 0.923 | 0.968 | 0.857 | 0.725 | 0.983 |
| GS_WDRVI | 0.956 | 0.998 | 0.996 | 0.956 | 0.967 | 0.956 | 0.956 | 0.998 |
| GS_WET | 0.983 | 0.983 | 0.983 | 0.983 | 0.983 | 0.983 | 0.983 | 0.754 |
| SS_ARVI | 0.525 | 0.882 | 0.691 | 0.541 | 0.535 | 0.674 | 0.534 | 0.882 |
| SS_BSI | 0.515 | 0.976 | 0.754 | 0.565 | 0.667 | 0.713 | 0.530 | 0.976 |
| SS_CI | 0.533 | 0.945 | 0.728 | 0.595 | 0.637 | 0.670 | 0.533 | 0.945 |
| SS_CVI | 0.633 | 0.985 | 0.813 | 0.685 | 0.633 | 0.643 | 0.686 | 0.985 |
| SS_EVI | 0.682 | 0.549 | 0.549 | 0.566 | 0.549 | 0.571 | 0.692 | 0.682 |
| SS_GLI | 0.541 | 0.849 | 0.652 | 0.541 | 0.679 | 0.695 | 0.570 | 0.849 |
| SS_GNDVI | 0.616 | 0.997 | 0.873 | 0.677 | 0.616 | 0.638 | 0.643 | 0.997 |
| SS_GVI | 0.506 | 0.929 | 0.892 | 0.622 | 0.686 | 0.699 | 0.540 | 0.929 |
| SS_GVMI | 0.600 | 0.825 | 0.895 | 0.600 | 0.628 | 0.782 | 0.605 | 0.825 |
| SS_mARI | 0.656 | 0.757 | 0.656 | 0.684 | 0.656 | 0.667 | 0.779 | 0.757 |
| SS_MAVI | 0.554 | 0.999 | 0.935 | 0.649 | 0.599 | 0.600 | 0.554 | 0.999 |
| SS_MCARI1 | 0.667 | 0.754 | 0.866 | 0.617 | 0.641 | 0.763 | 0.788 | 0.754 |
| SS_MSAVI | 0.554 | 0.825 | 0.917 | 0.636 | 0.605 | 0.554 | 0.585 | 0.825 |
| SS_NBR | 0.637 | 0.999 | 0.951 | 0.637 | 0.650 | 0.785 | 0.670 | 0.999 |
| SS_NBR2 | 0.532 | 0.751 | 0.657 | 0.589 | 0.542 | 0.558 | 0.527 | 0.751 |
| SS_NDBI | 0.637 | 0.999 | 0.951 | 0.637 | 0.650 | 0.785 | 0.670 | 0.999 |
| SS_NDMI | 0.624 | 0.997 | 0.915 | 0.624 | 0.655 | 0.771 | 0.649 | 0.997 |
| SS_NDSI | 0.624 | 0.997 | 0.915 | 0.624 | 0.655 | 0.771 | 0.649 | 0.997 |
| SS_NDVI | 0.558 | 0.999 | 0.932 | 0.653 | 0.598 | 0.610 | 0.558 | 0.999 |
| SS_NDWI | 0.616 | 0.997 | 0.873 | 0.677 | 0.616 | 0.638 | 0.643 | 0.997 |
| SS_RDI | 0.624 | 0.997 | 0.915 | 0.624 | 0.655 | 0.771 | 0.649 | 0.997 |
| SS_SAVI | 0.564 | 0.773 | 0.900 | 0.633 | 0.588 | 0.564 | 0.575 | 0.773 |
| SS_SBI | 0.505 | 0.898 | 0.836 | 0.601 | 0.664 | 0.692 | 0.533 | 0.898 |
| SS_SPVI | 0.562 | 0.915 | 0.931 | 0.661 | 0.629 | 0.549 | 0.649 | 0.915 |
| SS_TSAVI | 0.505 | 0.503 | 0.690 | 0.533 | 0.501 | 0.708 | 0.503 | 0.505 |
| SS_WDRVI | 0.558 | 0.999 | 0.932 | 0.653 | 0.598 | 0.610 | 0.558 | 0.999 |
| SS_WET | 0.508 | 0.929 | 0.882 | 0.595 | 0.677 | 0.723 | 0.517 | 0.929 |
| GS_blue | 0.958 | 0.989 | 0.990 | 0.958 | 0.958 | 0.958 | 0.959 | 0.989 |
| GS_green | 0.954 | 0.989 | 0.988 | 0.954 | 0.954 | 0.954 | 0.962 | 0.989 |
| GS_NIR | 0.890 | 0.890 | 0.890 | 0.890 | 0.890 | 0.890 | 0.929 | 0.808 |
| GS_red | 0.949 | 0.993 | 0.992 | 0.949 | 0.949 | 0.949 | 0.957 | 0.993 |
| GS_SWIR1 | 0.992 | 0.992 | 0.992 | 0.992 | 0.992 | 0.992 | 0.992 | 0.770 |
| GS_SWIR2 | 0.992 | 0.992 | 0.992 | 0.992 | 0.992 | 0.992 | 0.992 | 0.945 |
| SS_blue | 0.513 | 0.917 | 0.870 | 0.607 | 0.691 | 0.729 | 0.561 | 0.917 |
| SS_green | 0.533 | 0.913 | 0.858 | 0.616 | 0.662 | 0.626 | 0.565 | 0.913 |
| SS_NIR | 0.508 | 0.887 | 0.809 | 0.588 | 0.649 | 0.678 | 0.526 | 0.887 |
| SS_red | 0.509 | 0.913 | 0.854 | 0.601 | 0.662 | 0.665 | 0.525 | 0.913 |
| SS_SWIR1 | 0.585 | 0.585 | 0.635 | 0.592 | 0.585 | 0.585 | 0.630 | 0.537 |
| SS_SWIR2 | 0.592 | 0.592 | 0.592 | 0.602 | 0.592 | 0.592 | 0.627 | 0.537 |
| Aspect | 0.591 | 0.524 | 0.521 | 0.521 | 0.582 | 0.590 | 0.606 | 0.591 |
| CHM | 0.534 | 0.863 | 0.856 | 0.534 | 0.596 | 0.534 | 0.574 | 0.863 |
| Curvature | 0.543 | 0.511 | 0.511 | 0.511 | 0.511 | 0.516 | 0.561 | 0.543 |
| DAHI | 0.538 | 0.538 | 0.538 | 0.538 | 0.538 | 0.559 | 0.545 | 0.507 |
| Elevation | 0.886 | 0.999 | 0.984 | 0.886 | 0.952 | 0.886 | 0.979 | 0.999 |
| Roughness | 0.847 | 0.712 | 0.712 | 0.712 | 0.712 | 0.950 | 0.735 | 0.847 |
| Slope | 0.844 | 0.713 | 0.713 | 0.713 | 0.713 | 0.949 | 0.733 | 0.844 |
| TPI | 0.537 | 0.508 | 0.508 | 0.508 | 0.508 | 0.509 | 0.532 | 0.537 |
| TRI | 0.836 | 0.704 | 0.704 | 0.704 | 0.704 | 0.927 | 0.712 | 0.836 |
| TWI | 0.737 | 0.702 | 0.702 | 0.702 | 0.702 | 0.730 | 0.702 | 0.737 |


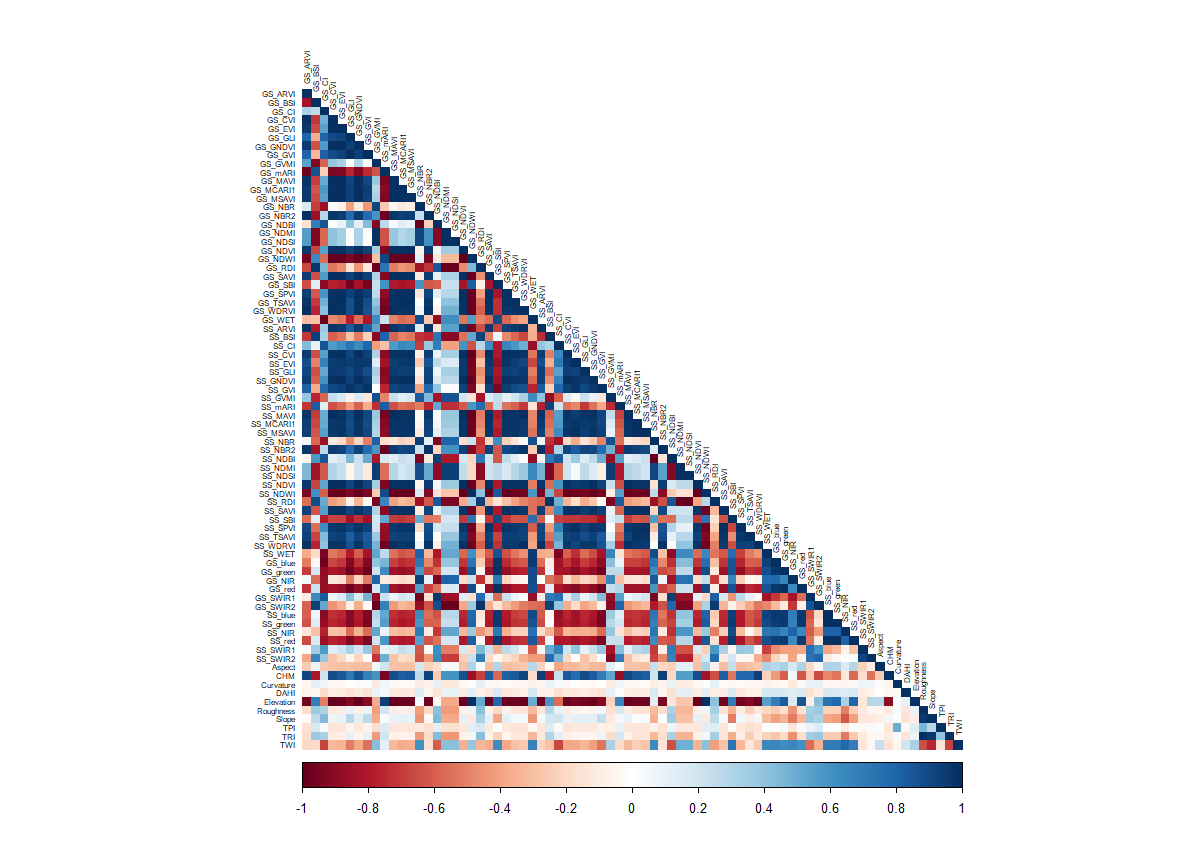


**Figure S4.** Correlogram of all the originally considered predictors, applied in 2009.

**Table S5.** Dimensions of the training dataset used to train the Level 1 RFs.

| **Class** | **Description** | **Average n. of pixels** | **SD (**±) |
| --- | --- | --- | --- |
| 1 | Rocks | 77697 | 3771 |
| 2 | Snow | 7083 | 406 |
| 3 | Water | 116 | 10 |
| 4 | Broadleaved | 5582 | 408 |
| 5 | Coniferous | 19393 | 1180 |
| 6 | Grassland | 39849 | 1827 |
| 7 | Shrubs | 6674 | 424 |
| 8 | Wetlands | 352 | 27 |


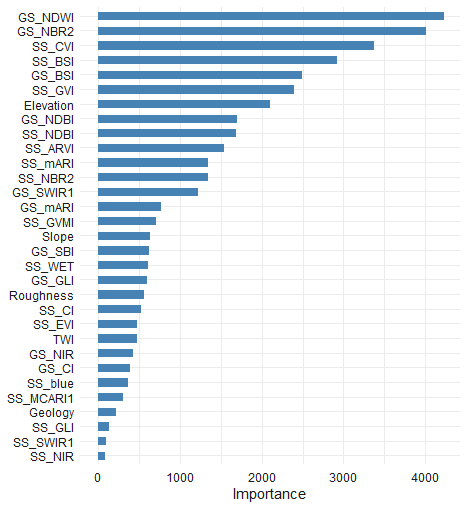


**Figure S5.** Variable importance plot for the RF built for 2009.

**Table S 6.** The 38 selected predictors: spectral indices and bands, computed both for the growing and the senescence seasons, and topographic and geological variables.

| **Spectral indices** | **Acronym** | **Formula** | **Specificity** | **Reference** |
| --- | --- | --- | --- | --- |
| Bare Soil Index | BSI | $\frac{\left( SWIR2+Red \right)-(NIR+Blue)}{\left( SWIR2+Red \right)+(NIR+Blue)}$ | Identifies bare soil areas and separates them from vegetation and urban areas. | Diek et al. (2017) |
| Coloration Index | CI | $\frac{Red-Blue}{Red}$ | Assesses the colour changes in vegetation, particularly in autumn leaves or stressed plants. | Escadafal et al. (1994) |
| Green Leaf Index | GLI | $\frac{2\times Green-Red-Blue}{2\times Green+Red+Blue}$ | Focuses on the green component of vegetation, indicating plant vitality. | Hunt et al. (2011) |
| Modified Anthocyanin Reflectance Index | mARI | $(\frac{1}{Green}-\frac{1}{Red})\times NIR$ | Estimates anthocyanin content, which is related to stress and senescence in plants. | Gitelson et al. (2006) |
| Normalized Burn Ratio 2 | NBR2 | $\frac{(SWIR1-SWIR2)}{(SWIR1+SWIR2)}$ | Identify burned area. Modifies the Normalized Burn Ratio (NBR) to highlight water sensitivity in vegetation. | U.S. Geological Survey Earth Resources Observation and Science Center (2016b) |
| Normalized Difference Built-up Index | NDBI | $\frac{(SWIR1-NIR)}{(SWIR1+NIR)}$ | Detects urban areas and built-up land. | Zha et al. (2003) |
| Normalized Difference Water Index | NDWI | $\frac{(Green-NIR)}{(Green+NIR)}$ | Monitors water content in vegetation and water bodies. | Gao (1996) |
| Tasselled Cap - Brightness | SBI | $0.3037\times Blue+0.2793\times Green+0.4743\times Red+0.5585\times NIR+0.5082\times SWIR1+0.1863\times SWIR2$ | Indicates the overall brightness of the land surface. | Bannari et al. (1995) |
| Tasselled Cap - Vegetation | GVI | $-0.2848\times Blue-0.2435\times Green-0.5436\times Red+0.7243\times NIR+0.084\times SWIR1-0.18\times SWIR2$ | Indicates vegetation greenness as part of the Tasselled Cap transformation. |  |
| Tasselled Cap - Wetness | WET | $0.1509\times Blue+0.1973\times Green+0.3279\times Red+0.3406\times NIR-0.7112\times SWIR1-0.4572\times SWIR2$ | Measures soil moisture content and vegetation wetness. |  |
| Atmospherically Resistant Vegetation Index | ARVI | $\frac{NIR-Red\times(Red-Blue)}{NIR+Red\times(Red-Blue)}$ | Reduces atmospheric interference, particularly aerosols, in vegetation monitoring. | Kaufman & Tanre (1992) |
| Chlorophyll Vegetation Index | CVI | $NIR\times(\frac{Red}{{Green}^{2}})$ | Estimates chlorophyll content in vegetation, focusing on plant health and productivity. | Glenn et al. (2010) |
| Enhanced Vegetation Index | EVI | $2.5\times(\frac{NIR-Red}{NIR+6\times Red-7.5\times Blue+1})$ | Improves sensitivity in high biomass regions and reduces atmospheric effects. | U.S. Geological Survey Earth Resources Observation and Science Center (2016a) |
| Global Vegetation Moisture Index | GVMI | $\frac{\left( NIR+0.1 \right)-(SWIR2+0.02)}{\left( NIR+0.1 \right)+(SWIR2+0.02)}$ | Estimates vegetation moisture content, useful in drought monitoring. | Ceccato et al. (2002) |
| Modified Chlorophyll Absorption in Reflectance Index 1 | MCARI1 | $1.2\times(2.5\times\left( NIR-Red \right)-1.3\times\left( NIR-Green \right))$ | Estimates chlorophyll concentration while minimizing soil background effects. | Haboudane et al. (2004) |
| **Spectral bands** | **Acronym** | **L4-5 (TM-MSS) (µm)** | **L7 (TM+) (µm)** | **L8-9 (OLI/OLI+) (µm)** |
| Near Infrared | NIR | 0.76– 0.90 | 0.77 – 0.90 | 0.85 – 0.88 |
| Shortwave Infrared 1 | SWIR1 | 1.55 – 1.75 | 1.55 – 1.75 | 1.57 – 1.65 |
| Blue | Blue | 0.45 – 0.52 | 0.45 – 0.52 | 0.45 – 0.51 |
| **Topography** | **Resolution** | **Unit** | **Specificity** | **Source** |
| Elevation | 5 m | Meters | Altitude exerts a strong control over zonal vegetation distribution. | Derived from the DTMs distributed by the Piedmont and Aosta Valley Regions geoportals |
| Roughness | 5 m | Degrees | Roughness represents micro-topographical features. |  |
| Slope | 5 m | Percentage | Represents the steepness or incline of a surface. |  |
| Topographic Wetness Index (TWI) | 5 m | Dimensionless | TWI is a measure of the potential for water to accumulate in a specific area based on the surrounding terrain. |  |
| Geology | 1:100.000 | Geological units | Spatial distribution of geological features, including rock types, formations, and structural attributes, across the study area. | Geological Map of Italy scale 1:100,000, ISPRA |

**Table S7.** Available orthoimagery used for building the independent validation dataset. BN = black & white; RGB = Red, Green, Blue.

| **Year** | **Type** | **Coverage** | **Property** | **Source (accessed on 04/05/2022)** |
| --- | --- | --- | --- | --- |
| 1988 | BN | GPNP | Ministero dell'Ambiente e della Tutela del Territorio e del Mare - Geoportale nazionale | http://www.pcn.minambiente.it/geoportal/rest/document?id=m_amte%3A299FN3%3Aa1197b44-5b11-4a49-def8-ae45e2c5e6ae |
| 1991 | BN | Piedmont side | Regione Piemonte | http://www.geoportale.piemonte.it/geocatalogorp/index.jsp |
| 1994 | BN | GPNP | Agenzia per le erogazioni in agricoltura | http://www.pcn.minambiente.it/geoportal/rest/document?id=m_amte%3A299FN3%3A4dca3621-4baa-49ee-90b5-33ca42a4bb4e |
| 2000 | RGB | GPNP | BLOM CGR S.P.A. | http://www.pcn.minambiente.it/geoportal/rest/document?id=m_amte%3A299FN3%3A2637cb18-6d0c-4508-bd6e-64804e85a550 |
| 2005 | RGB | GPNP | Arpa Piemonte | http://www.geoportale.piemonte.it/geonetworkrp/srv/ita/metadata.show?id=5862&currTab=rndt |
| 2006 | RGB | GPNP | BLOM CGR S.P.A. | http://www.pcn.minambiente.it/geoportal/rest/document?id=m_amte%3A299FN3%3A21d09438-3f4c-4642-a9ea-064a60488b88 |
| 2010 | RGB | Piedmont side | Regione Piemonte | http://www.geoportale.piemonte.it/geonetworkrp/srv/ita/metadata.show?id=2557&currTab=rndt |
| 2012 | RGB | GPNP | Agenzia per le erogazioni in agricoltura | http://www.pcn.minambiente.it/geoportal/rest/document?id=m_amte%3A299FN3%3Aeda6494c-b619-441a-a2d7-4097f8cde540 |
| 2015 | RGB | Piedmont side | Consorzio TeA | http://www.geoportale.piemonte.it/geonetworkrp/srv/ita/metadata.show?id=6626&currTab=rndt |
| 2018 | RGB | Piedmont side | AGEA 2018 (c) | http://www.geoportale.piemonte.it/geonetworkrp/srv/ita/metadata.show?id=7167&currTab=rndt |

**Table S 8** Overall scores metrics. K = Cohen’s K coefficient; OA = Overall Accuracy; WA = Weighted Accuracy.

| **Year** |  | **OA** | **K** | **WA** |
| --- | --- | --- | --- | --- |
| 1985 |  | 0.99 | 0.99 | 0.99 |
| 1990 |  | 0.99 | 0.98 | 0.99 |
| 1995 |  | 0.97 | 0.96 | 0.98 |
| 2000 |  | 0.96 | 0.95 | 0.96 |
| 2005 |  | 0.98 | 0.98 | 0.98 |
| 2010 |  | 0.95 | 0.94 | 0.95 |
| 2015 |  | 0.98 | 0.97 | 0.98 |
| 2020 |  | 0.98 | 0.97 | 0.98 |


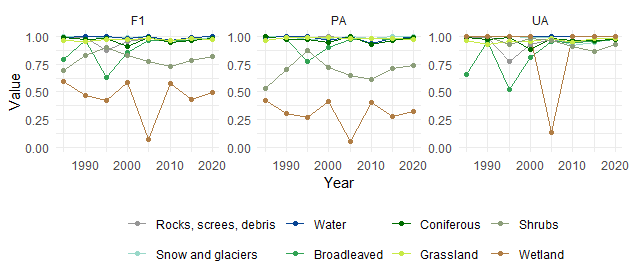


***Figure S* 6*.*** *Class metrics for intermediate annual maps*, before applying post-processing*. F1 = F-1 score; PA = Producer’s Accuracy; UA = User’s Accuracy.*
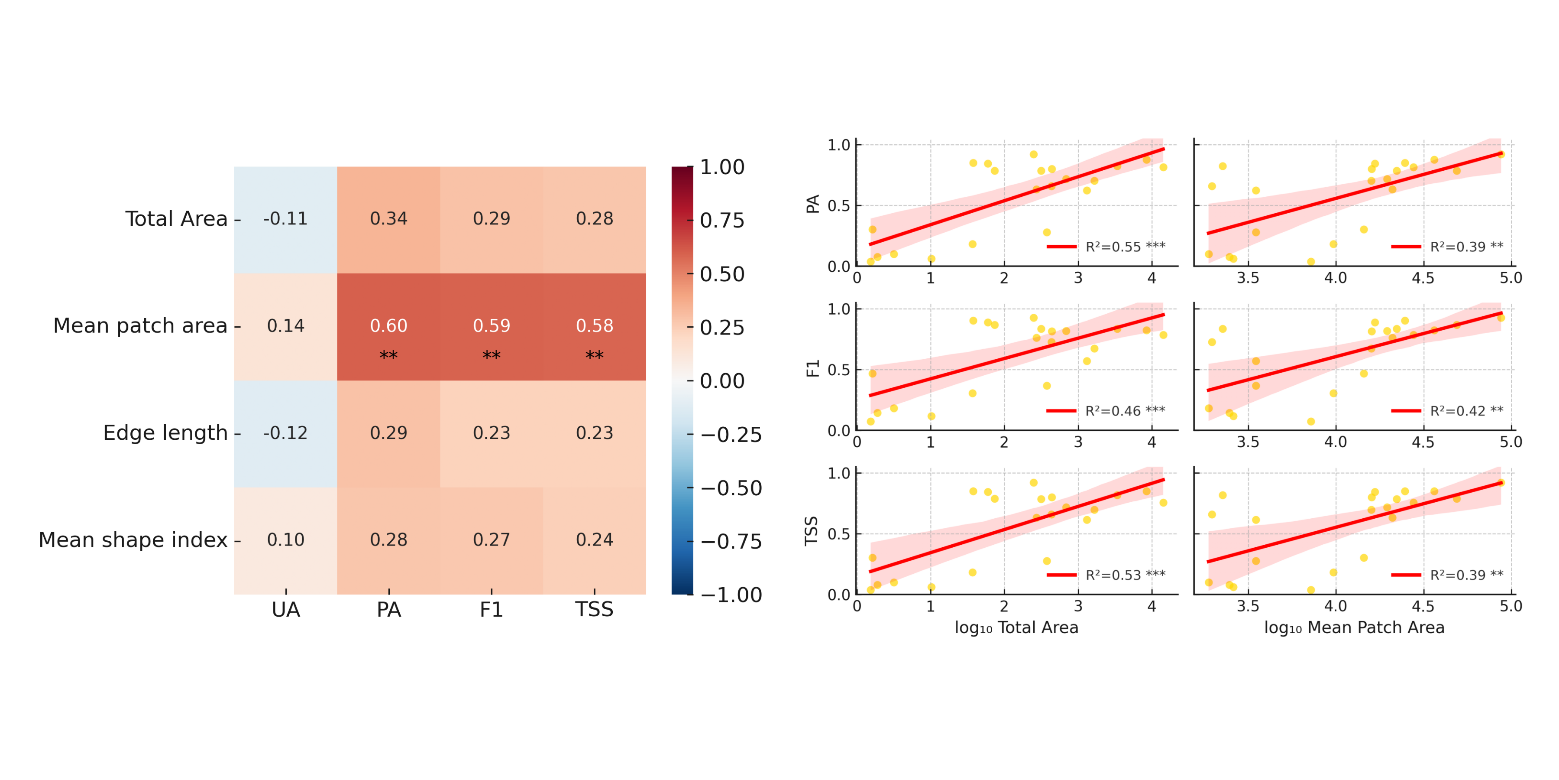


**Figure S 7.** Relationship between structural metrics of habitat patches and classification accuracy. Significance level ** = p < 0.01.


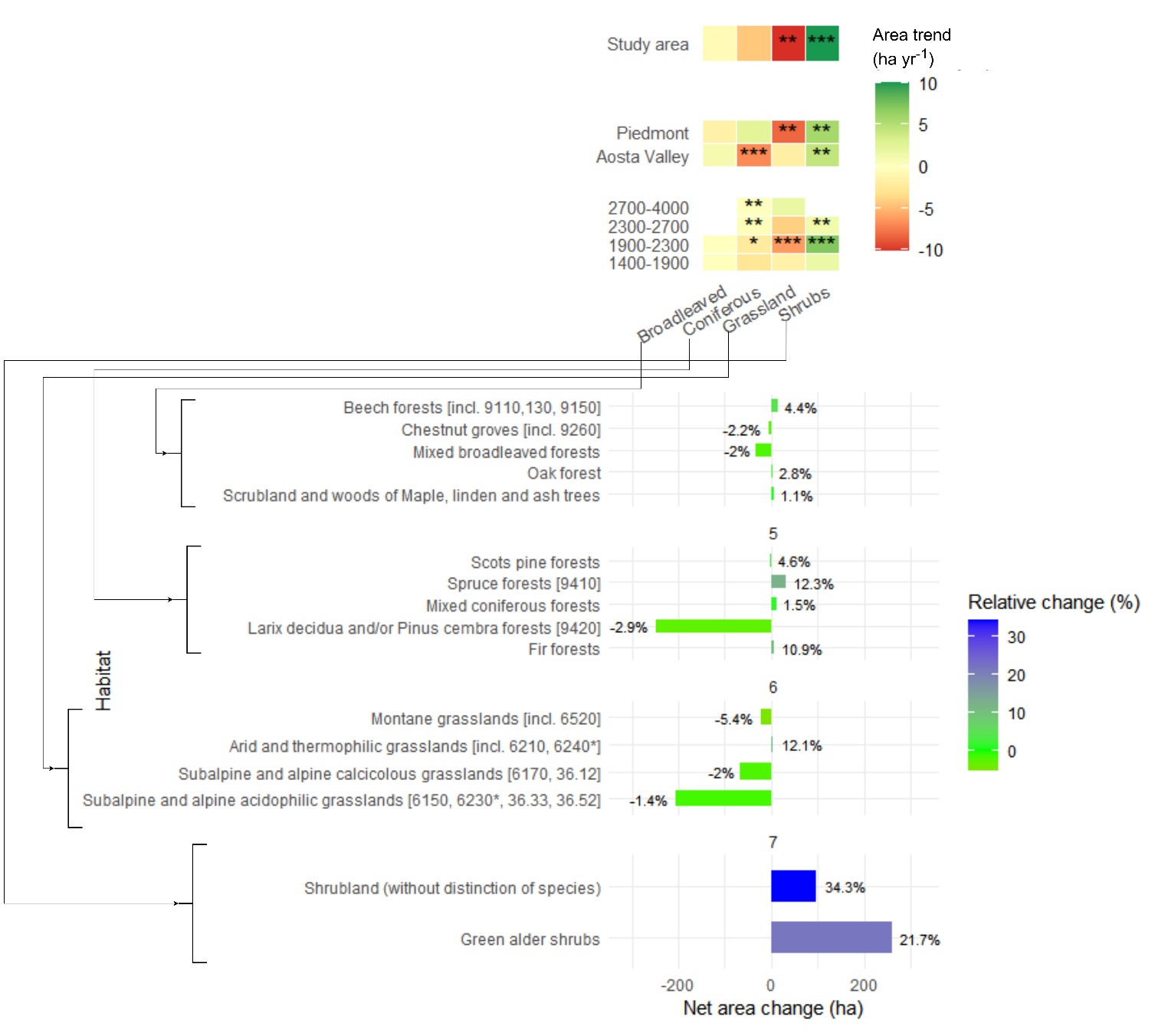


**Figure S 8.** Upper panel: trends of the different land cover classes, or time rate of change, in loss or gain, with significance stars (* p < 0.05, ** p < 0.01, *** p < 0.001) for the overall study area, Region and elevation (m a.s.l.). Lower panel: habitats net (total hectares of gain or loss) and relative (% of variation compared to the initial extent) change.

*
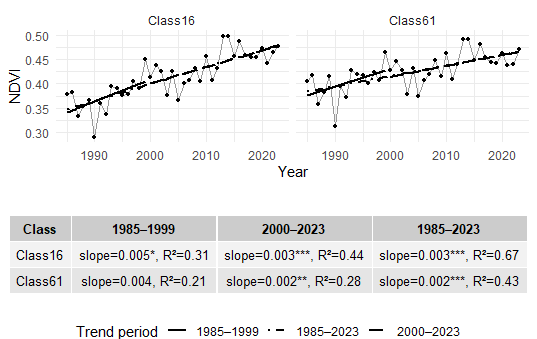
*

**Figure S 9**. NDVI trends for Rocks to Grassland (Class 16) and Grassland to Rocks (Class 61) transition pixels.


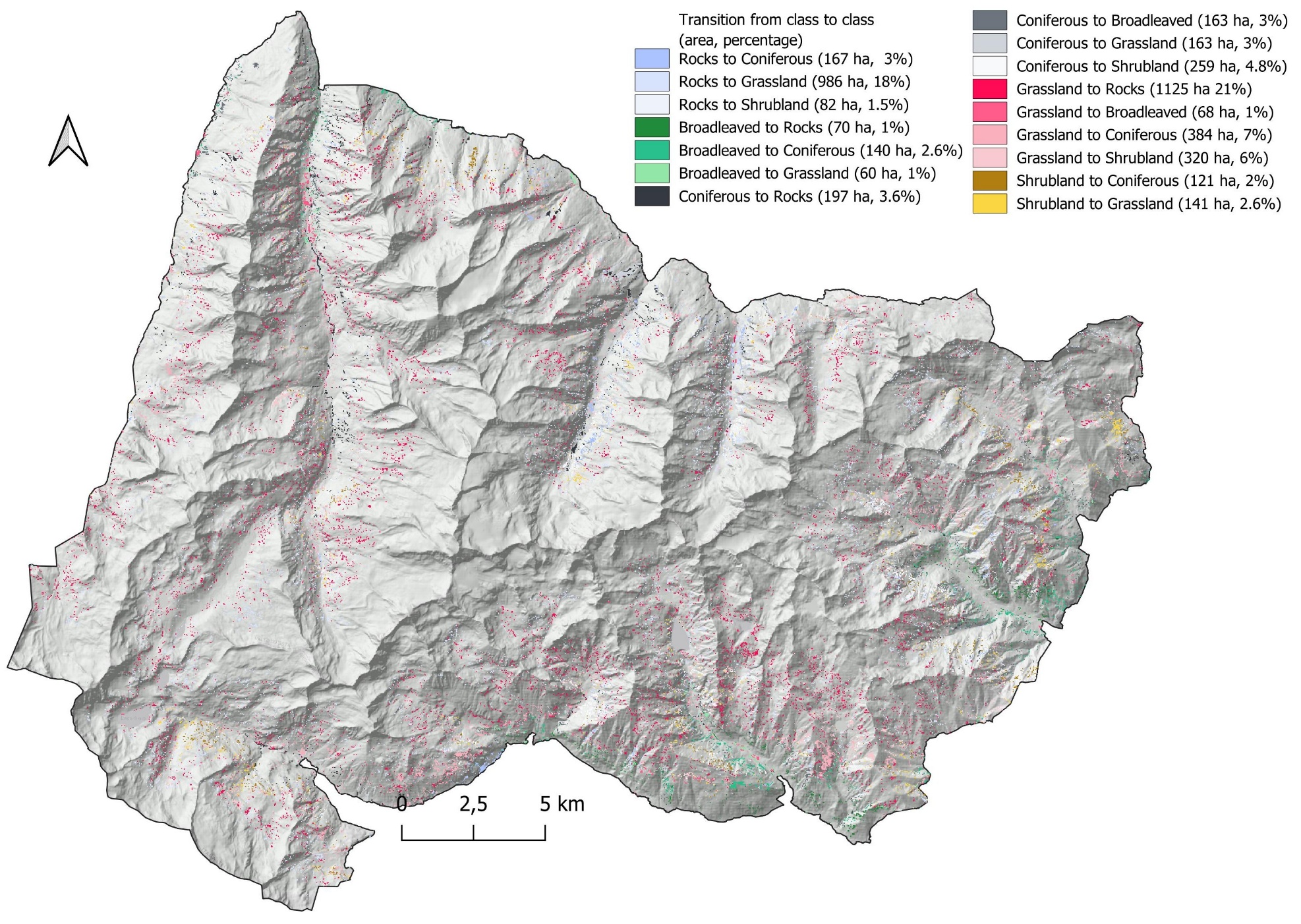


**Figure S 10.** Land cover transitions from 1985 to 2023. Transitions lower than 1% were excluded.


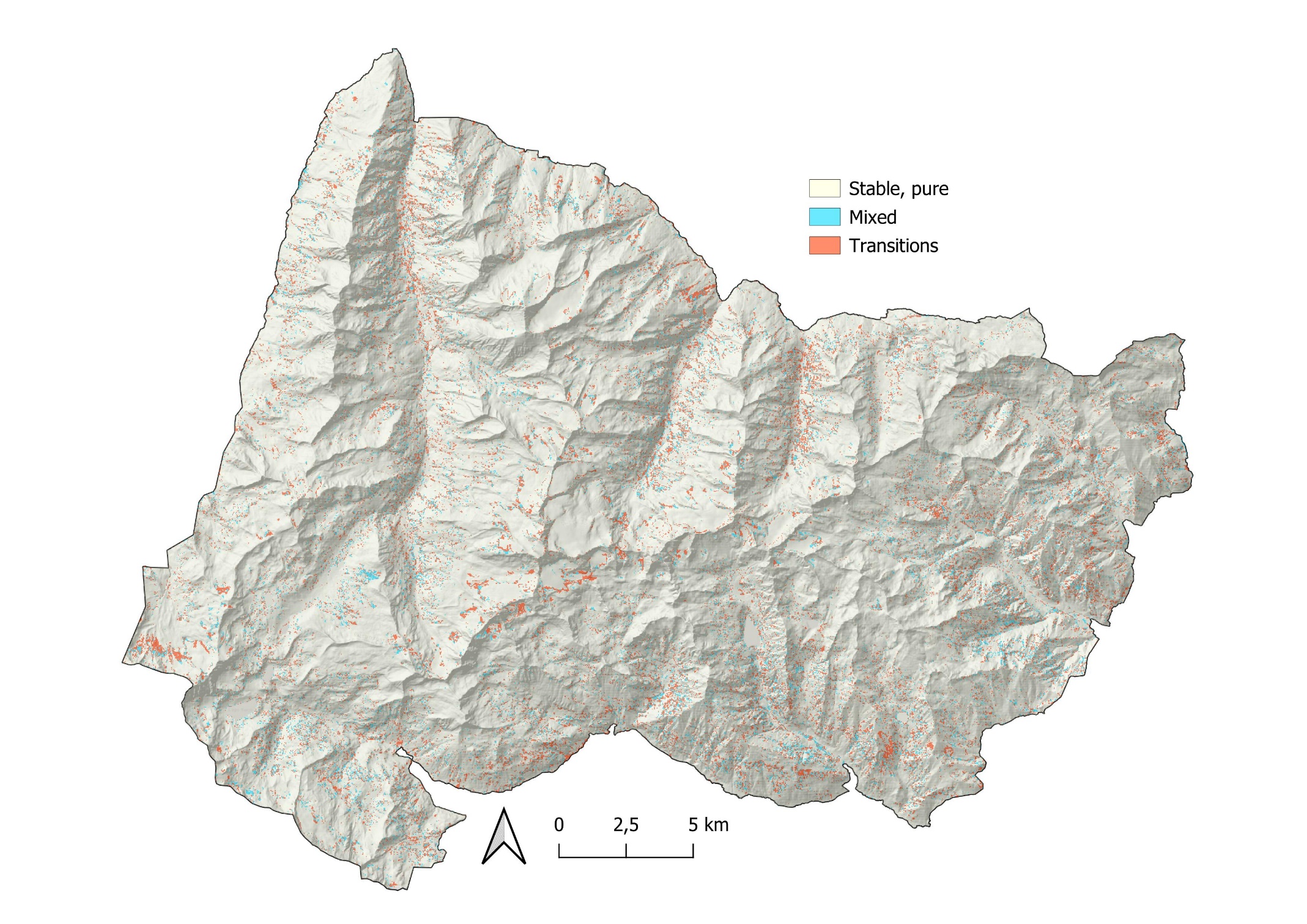


**Figure S 11**. Map of stable–mixed and transition areas from 1985 to 2023.

**Table S 9.** Full list of Landsat scene IDs used in the analysis, indicating the year and season in which they were used for the BAP compositing.

| Year | Season | Scene id |
| --- | --- | --- |
| 1985 | GS | LT05_L2SP_195028_19850710_20200918_02_T1 |
|  |  | LT05_L2SP_195028_19850811_20200918_02_T1 |
|  |  | LT05_L2SP_195028_19850827_20200918_02_T1 |
|  | SS | LT05_L2SP_195028_19851115_20200918_02_T1 |
| 1986 | GS | LT05_L2SP_195028_19860729_20200918_02_T1 |
|  |  | LT05_L2SP_195028_19860814_20200918_02_T1 |
|  | SS | LT05_L2SP_195028_19861017_20200917_02_T1 |
|  |  | LT05_L2SP_195028_19861118_20200917_02_T1 |
| 1987 | GS | LT05_L2SP_195028_19870630_20201014_02_T1 |
|  |  | LT05_L2SP_195028_19870801_20201014_02_T1 |
|  |  | LT05_L2SP_195028_19870817_20201014_02_T1 |
|  | SS | LT05_L2SP_195028_19870918_20211115_02_T1 |
|  |  | LT05_L2SP_195028_19871020_20201014_02_T1 |
|  |  | LT05_L2SP_195028_19871105_20201014_02_T1 |
|  |  | LT05_L2SP_195028_19871121_20201014_02_T1 |
| 1988 | GS | LT04_L2SP_195028_19880811_20201008_02_T1 |
|  |  | LT04_L2SP_195028_19880827_20200917_02_T1 |
|  |  | LT05_L2SP_195028_19880616_20211118_02_T1 |
|  |  | LT05_L2SP_195028_19880702_20200917_02_T1 |
|  |  | LT05_L2SP_195028_19880718_20200917_02_T1 |
|  |  | LT05_L2SP_195028_19880803_20200917_02_T1 |
|  | SS | LT05_L2SP_195028_19880920_20211118_02_T1 |
|  |  | LT05_L2SP_195028_19881006_20200917_02_T1 |
|  |  | LT05_L2SP_195028_19881022_20211118_02_T1 |
| 1989 | GS | LT05_L2SP_195028_19890705_20200916_02_T1 |
|  |  | LT05_L2SP_195028_19890721_20200916_02_T1 |
|  |  | LT05_L2SP_195028_19890822_20200916_02_T1 |
|  | SS | LT05_L2SP_195028_19891009_20200916_02_T1 |
| 1990 | GS | LT04_L2SP_195028_19900716_20200916_02_T1 |
|  |  | LT05_L2SP_195028_19900809_20211121_02_T1 |
|  |  | LT05_L2SP_195028_19900825_20200915_02_T1 |
|  | SS | LT05_L2SP_195028_19900926_20200915_02_T1 |
|  |  | LT05_L2SP_195028_19901012_20200915_02_T1 |
|  |  | LT05_L2SP_195028_19901028_20200915_02_T1 |
| 1991 | GS | LT05_L2SP_195028_19910625_20200915_02_T1 |
|  |  | LT05_L2SP_195028_19910711_20200915_02_T1 |
|  |  | LT05_L2SP_195028_19910727_20200915_02_T1 |
|  |  | LT05_L2SP_195028_19910812_20200915_02_T1 |
|  | SS | LT05_L2SP_195028_19911015_20200915_02_T1 |
| 1992 | GS | LT05_L2SP_195028_19920627_20200914_02_T1 |
|  |  | LT05_L2SP_195028_19920814_20200914_02_T1 |
|  |  | LT05_L2SP_195028_19920830_20200914_02_T1 |
|  | SS | LT05_L2SP_195028_19920915_20200914_02_T1 |
|  |  | LT05_L2SP_195028_19921001_20200914_02_T1 |
|  |  | LT05_L2SP_195028_19921102_20200914_02_T1 |
| 1993 | GS | LT05_L2SP_195028_19930716_20200913_02_T1 |
|  |  | LT05_L2SP_195028_19930801_20200913_02_T1 |
|  |  | LT05_L2SP_195028_19930817_20200913_02_T1 |
|  | SS | LT05_L2SP_195028_19931004_20200913_02_T1 |
|  |  | LT05_L2SP_195028_19931020_20200913_02_T1 |
| 1994 | GS | LT05_L2SP_195028_19940617_20200913_02_T1 |
|  |  | LT05_L2SP_195028_19940703_20200913_02_T1 |
|  |  | LT05_L2SP_195028_19940804_20200913_02_T1 |
|  |  | LT05_L2SP_195028_19940820_20200913_02_T1 |
|  | SS | LT05_L2SP_195028_19941108_20200912_02_T1 |
| 1995 | GS | LT05_L2SP_195028_19950620_20200912_02_T1 |
|  |  | LT05_L2SP_195028_19950706_20200912_02_T1 |
|  |  | LT05_L2SP_195028_19950722_20200912_02_T1 |
|  | SS | LT05_L2SP_195028_19951010_20211122_02_T1 |
|  |  | LT05_L2SP_195028_19951026_20200912_02_T1 |
| 1996 | GS | LT05_L2SP_195028_19960809_20200911_02_T1 |
|  |  | LT05_L2SP_195028_19960825_20200911_02_T1 |
|  | SS | LT05_L2SP_195028_19960926_20200911_02_T1 |
|  |  | LT05_L2SP_195028_19961012_20211124_02_T1 |
|  |  | LT05_L2SP_195028_19961028_20211124_02_T1 |
|  |  | LT05_L2SP_195028_19961129_20211124_02_T1 |
| 1997 | GS | LT05_L2SP_195028_19970727_20200910_02_T1 |
|  |  | LT05_L2SP_195028_19970812_20200910_02_T1 |
|  | SS | LT05_L2SP_195028_19970929_20200909_02_T1 |
|  |  | LT05_L2SP_195028_19971031_20211128_02_T1 |
|  |  | LT05_L2SP_195028_19971116_20211129_02_T1 |
| 1998 | GS | LT05_L2SP_195028_19980628_20200909_02_T1 |
|  |  | LT05_L2SP_195028_19980714_20200909_02_T1 |
|  |  | LT05_L2SP_195028_19980730_20200908_02_T1 |
|  |  | LT05_L2SP_195028_19980815_20200908_02_T1 |
|  |  | LT05_L2SP_195028_19980831_20200908_02_T1 |
|  | SS | LT05_L2SP_195028_19980916_20200908_02_T1 |
|  |  | LT05_L2SP_195028_19981002_20211129_02_T1 |
| 1999 | GS | LE07_L2SP_195028_19990725_20200918_02_T1 |
|  |  | LE07_L2SP_195028_19990810_20200918_02_T1 |
|  |  | LT05_L2SP_195028_19990717_20200907_02_T1 |
|  |  | LT05_L2SP_195028_19990802_20211205_02_T1 |
|  | SS | LE07_L2SP_195028_19991029_20211205_02_T1 |
|  |  | LE07_L2SP_195028_19991130_20200918_02_T1 |
|  |  | LT05_L2SP_195028_19991005_20200907_02_T1 |
| 2000 | GS | LE07_L2SP_195028_20000625_20200918_02_T1 |
|  |  | LE07_L2SP_195028_20000727_20200918_02_T1 |
|  |  | LE07_L2SP_195028_20000828_20211120_02_T1 |
|  |  | LT05_L2SP_195028_20000719_20200906_02_T1 |
|  |  | LT05_L2SP_195028_20000820_20200906_02_T1 |
|  | SS | LE07_L2SP_195028_20000929_20200917_02_T1 |
|  |  | LE07_L2SP_195028_20001015_20211214_02_T1 |
|  |  | LT05_L2SP_195028_20000921_20200906_02_T1 |
| 2001 | GS | LE07_L2SP_195028_20010730_20200917_02_T1 |
|  |  | LE07_L2SP_195028_20010815_20211127_02_T1 |
|  |  | LT05_L2SP_195028_20010722_20200906_02_T1 |
|  |  | LT05_L2SP_195028_20010823_20200905_02_T1 |
|  | SS | LE07_L2SP_195028_20010916_20200917_02_T1 |
|  |  | LE07_L2SP_195028_20011002_20200917_02_T1 |
|  |  | LE07_L2SP_195028_20011103_20200917_02_T1 |
|  |  | LE07_L2SP_195028_20011119_20200917_02_T1 |
| 2002 | GS | LE07_L2SP_195028_20020701_20211129_02_T1 |
|  |  | LE07_L2SP_195028_20020802_20200916_02_T1 |
|  |  | LE07_L2SP_195028_20020818_20200916_02_T1 |
|  |  | LT05_L2SP_195028_20020623_20200905_02_T1 |
|  | SS | LE07_L2SP_195028_20021005_20200916_02_T1 |
|  |  | LE07_L2SP_195028_20021106_20200916_02_T1 |
|  |  | LE07_L2SP_195028_20021122_20211202_02_T1 |
| 2003 | GS | LE07_L2SP_195028_20030805_20200916_02_T1 |
|  |  | LT05_L2SP_195028_20030712_20200904_02_T1 |
|  |  | LT05_L2SP_195028_20030813_20200904_02_T1 |
|  | SS | LE07_L2SP_195028_20031109_20200915_02_T1 |
|  |  | LE07_L2SP_195028_20031125_20200915_02_T1 |
|  |  | LT05_L2SP_195028_20030930_20200904_02_T1 |
|  |  | LT05_L2SP_195028_20031016_20200904_02_T1 |
| 2004 | GS | LE07_L2SP_195028_20040722_20200915_02_T1 |
|  |  | LE07_L2SP_195028_20040823_20200915_02_T1 |
|  |  | LT05_L2SP_195028_20040714_20201008_02_T1 |
|  |  | LT05_L2SP_195028_20040730_20201008_02_T1 |
|  |  | LT05_L2SP_195028_20040831_20200903_02_T1 |
|  | SS | LE07_L2SP_195028_20041010_20200915_02_T1 |
|  |  | LT05_L2SP_195028_20041002_20200903_02_T1 |
| 2005 | GS | LE07_L2SP_195028_20050623_20200914_02_T1 |
|  |  | LE07_L2SP_195028_20050810_20200914_02_T1 |
|  |  | LT05_L2SP_195028_20050615_20200902_02_T1 |
|  |  | LT05_L2SP_195028_20050701_20200902_02_T1 |
|  |  | LT05_L2SP_195028_20050818_20200902_02_T1 |
|  | SS | LE07_L2SP_195028_20051013_20200914_02_T1 |
|  |  | LE07_L2SP_195028_20051029_20200914_02_T1 |
|  |  | LE07_L2SP_195028_20051114_20200914_02_T1 |
|  |  | LT05_L2SP_195028_20051106_20200901_02_T1 |
|  |  | LT05_L2SP_195028_20051122_20201008_02_T1 |
| 2006 | GS | LE07_L2SP_195028_20060626_20200914_02_T1 |
|  |  | LE07_L2SP_195028_20060712_20200914_02_T1 |
|  |  | LT05_L2SP_195028_20060720_20200831_02_T1 |
|  |  | LT05_L2SP_195028_20060821_20200831_02_T1 |
|  | SS | LE07_L2SP_195028_20061016_20200913_02_T1 |
|  |  | LE07_L2SP_195028_20061101_20200913_02_T1 |
|  |  | LT05_L2SP_195028_20060922_20200831_02_T1 |
| 2007 | GS | LE07_L2SP_195028_20070715_20200913_02_T1 |
|  |  | LE07_L2SP_195028_20070731_20200913_02_T1 |
|  |  | LT05_L2SP_195028_20070621_20211209_02_T1 |
|  |  | LT05_L2SP_195028_20070824_20200830_02_T1 |
|  | SS | LE07_L2SP_195028_20071019_20200913_02_T1 |
|  |  | LE07_L2SP_195028_20071104_20200913_02_T1 |
| 2008 | GS | LE07_L2SP_195028_20080701_20200912_02_T1 |
|  |  | LE07_L2SP_195028_20080818_20200913_02_T1 |
|  |  | LT05_L2SP_195028_20080623_20200829_02_T1 |
|  |  | LT05_L2SP_195028_20080725_20200829_02_T1 |
|  |  | LT05_L2SP_195028_20080826_20200829_02_T1 |
|  | SS | LE07_L2SP_195028_20080919_20200912_02_T1 |
|  |  | LE07_L2SP_195028_20081005_20200913_02_T1 |
|  |  | LE07_L2SP_195028_20081106_20200912_02_T1 |
| 2009 | GS | LE07_L2SP_195028_20090618_20200912_02_T1 |
|  |  | LE07_L2SP_195028_20090720_20200911_02_T1 |
|  |  | LT05_L2SP_195028_20090728_20200827_02_T1 |
|  |  | LT05_L2SP_195028_20090829_20200825_02_T1 |
|  | SS | LE07_L2SP_195028_20090922_20200911_02_T1 |
|  |  | LT05_L2SP_195028_20090930_20200825_02_T1 |
|  |  | LT05_L2SP_195028_20091016_20200825_02_T1 |
|  |  | LT05_L2SP_195028_20091101_20201008_02_T1 |
| 2010 | GS | LE07_L2SP_195028_20100707_20200911_02_T1 |
|  |  | LE07_L2SP_195028_20100808_20200911_02_T1 |
|  |  | LT05_L2SP_195028_20100629_20200823_02_T1 |
|  |  | LT05_L2SP_195028_20100731_20200824_02_T1 |
|  | SS | LE07_L2SP_195028_20101027_20200910_02_T1 |
|  |  | LT05_L2SP_195028_20101019_20200823_02_T1 |
|  |  | LT05_L2SP_195028_20101104_20200823_02_T1 |
|  |  | LT05_L2SP_195028_20101120_20200823_02_T1 |
| 2011 | GS | LE07_L2SP_195028_20110624_20200910_02_T1 |
|  |  | LE07_L2SP_195028_20110811_20200910_02_T1 |
|  |  | LE07_L2SP_195028_20110827_20200910_02_T1 |
|  |  | LT05_L2SP_195028_20110616_20200822_02_T1 |
|  |  | LT05_L2SP_195028_20110702_20200822_02_T1 |
|  | SS | LE07_L2SP_195028_20110928_20200909_02_T1 |
|  |  | LE07_L2SP_195028_20111014_20200909_02_T1 |
|  |  | LE07_L2SP_195028_20111030_20200909_02_T1 |
|  |  | LE07_L2SP_195028_20111115_20200909_02_T1 |
|  |  | LT05_L2SP_195028_20110920_20200820_02_T1 |
|  |  | LT05_L2SP_195028_20111006_20200820_02_T1 |
|  |  | LT05_L2SP_195028_20111022_20200820_02_T1 |
|  |  | LT05_L2SP_195028_20111107_20200820_02_T1 |
| 2012 | GS | LE07_L2SP_195028_20120712_20200908_02_T1 |
|  | SS | LE07_L2SP_195028_20121016_20200908_02_T1 |
|  |  | LE07_L2SP_195028_20121101_20200908_02_T1 |
|  |  | LE07_L2SP_195028_20121117_20200908_02_T1 |
| 2013 | GS | LC08_L2SP_195028_20130621_20200912_02_T1 |
|  |  | LC08_L2SP_195028_20130707_20200912_02_T1 |
|  |  | LC08_L2SP_195028_20130723_20200912_02_T1 |
|  |  | LC08_L2SP_195028_20130824_20200913_02_T1 |
|  |  | LE07_L2SP_195028_20130715_20200907_02_T1 |
|  |  | LE07_L2SP_195028_20130731_20200907_02_T1 |
|  |  | LE07_L2SP_195028_20130816_20200907_02_T1 |
|  | SS | LC08_L2SP_195028_20130925_20200913_02_T1 |
|  |  | LC08_L2SP_195028_20131112_20200912_02_T1 |
|  |  | LC08_L2SP_195028_20131128_20200912_02_T1 |
|  |  | LE07_L2SP_195028_20131019_20200907_02_T1 |
| 2014 | GS | LC08_L2SP_195028_20140624_20200911_02_T1 |
|  |  | LC08_L2SP_195028_20140827_20200911_02_T1 |
|  |  | LE07_L2SP_195028_20140718_20200906_02_T1 |
|  | SS | LC08_L2SP_195028_20140928_20200911_02_T1 |
|  |  | LC08_L2SP_195028_20141014_20200911_02_T1 |
|  |  | LC08_L2SP_195028_20141030_20200910_02_T1 |
|  |  | LE07_L2SP_195028_20141006_20200905_02_T1 |
| 2015 | GS | LC08_L2SP_195028_20150627_20200909_02_T1 |
|  |  | LC08_L2SP_195028_20150713_20200909_02_T1 |
|  |  | LC08_L2SP_195028_20150814_20200908_02_T1 |
|  |  | LC08_L2SP_195028_20150830_20200908_02_T1 |
|  |  | LE07_L2SP_195028_20150619_20200904_02_T1 |
|  |  | LE07_L2SP_195028_20150705_20200904_02_T1 |
|  |  | LE07_L2SP_195028_20150721_20200904_02_T1 |
|  |  | LE07_L2SP_195028_20150806_20200904_02_T1 |
|  |  | LE07_L2SP_195028_20150822_20200904_02_T1 |
|  | SS | LC08_L2SP_195028_20150915_20200908_02_T1 |
|  |  | LC08_L2SP_195028_20151017_20200908_02_T1 |
|  |  | LC08_L2SP_195028_20151102_20210219_02_T1 |
|  |  | LC08_L2SP_195028_20151118_20200908_02_T1 |
|  |  | LE07_L2SP_195028_20150923_20200903_02_T1 |
|  |  | LE07_L2SP_195028_20151025_20200903_02_T1 |
|  |  | LE07_L2SP_195028_20151110_20200903_02_T1 |
|  |  | LE07_L2SP_195028_20151126_20200903_02_T1 |
| 2016 | GS | LC08_L2SP_195028_20160629_20200906_02_T1 |
|  |  | LC08_L2SP_195028_20160715_20200906_02_T1 |
|  |  | LC08_L2SP_195028_20160816_20200906_02_T1 |
|  |  | LE07_L2SP_195028_20160707_20200902_02_T1 |
|  |  | LE07_L2SP_195028_20160723_20200902_02_T1 |
|  |  | LE07_L2SP_195028_20160808_20200902_02_T1 |
|  |  | LE07_L2SP_195028_20160824_20200902_02_T1 |
|  | SS | LC08_L2SP_195028_20161003_20200906_02_T1 |
|  |  | LC08_L2SP_195028_20161019_20200905_02_T1 |
|  |  | LC08_L2SP_195028_20161104_20200905_02_T1 |
|  |  | LC08_L2SP_195028_20161120_20200905_02_T1 |
|  |  | LE07_L2SP_195028_20160925_20200902_02_T1 |
|  |  | LE07_L2SP_195028_20161011_20200901_02_T1 |
|  |  | LE07_L2SP_195028_20161027_20200901_02_T1 |
|  |  | LE07_L2SP_195028_20161112_20200901_02_T1 |
|  |  | LE07_L2SP_195028_20161128_20200901_02_T1 |
| 2017 | GS | LC08_L2SP_195028_20170616_20200903_02_T1 |
|  |  | LC08_L2SP_195028_20170702_20200903_02_T1 |
|  |  | LC08_L2SP_195028_20170803_20200903_02_T1 |
|  |  | LC08_L2SP_195028_20170819_20200903_02_T1 |
|  |  | LE07_L2SP_195028_20170624_20200831_02_T1 |
|  |  | LE07_L2SP_195028_20170726_20200831_02_T1 |
|  |  | LE07_L2SP_195028_20170827_20200831_02_T1 |
|  | SS | LC08_L2SP_195028_20170920_20200903_02_T1 |
|  |  | LC08_L2SP_195028_20171006_20200903_02_T1 |
|  |  | LC08_L2SP_195028_20171022_20200902_02_T1 |
|  |  | LC08_L2SP_195028_20171107_20200902_02_T1 |
|  |  | LC08_L2SP_195028_20171123_20200902_02_T1 |
|  |  | LE07_L2SP_195028_20170928_20200830_02_T1 |
|  |  | LE07_L2SP_195028_20171014_20200830_02_T1 |
|  |  | LE07_L2SP_195028_20171030_20200830_02_T1 |
|  |  | LE07_L2SP_195028_20171115_20200830_02_T1 |
| 2018 | GS | LC08_L2SP_195028_20180619_20200831_02_T1 |
|  |  | LC08_L2SP_195028_20180806_20200831_02_T1 |
|  |  | LE07_L2SP_195028_20180627_20200829_02_T1 |
|  |  | LE07_L2SP_195028_20180713_20200829_02_T1 |
|  |  | LE07_L2SP_195028_20180729_20200829_02_T1 |
|  |  | LE07_L2SP_195028_20180830_20200828_02_T1 |
|  | SS | LC08_L2SP_195028_20180923_20200830_02_T1 |
|  |  | LC08_L2SP_195028_20181009_20200830_02_T1 |
|  |  | LC08_L2SP_195028_20181025_20200830_02_T1 |
|  |  | LC08_L2SP_195028_20181110_20200830_02_T1 |
|  |  | LE07_L2SP_195028_20180915_20200828_02_T1 |
|  |  | LE07_L2SP_195028_20181017_20200828_02_T1 |
|  |  | LE07_L2SP_195028_20181118_20200827_02_T1 |
| 2019 | GS | LC08_L2SP_195028_20190708_20200827_02_T1 |
|  |  | LC08_L2SP_195028_20190724_20200827_02_T1 |
|  |  | LC08_L2SP_195028_20190809_20200827_02_T1 |
|  |  | LC08_L2SP_195028_20190825_20200826_02_T1 |
|  |  | LE07_L2SP_195028_20190630_20200825_02_T1 |
|  |  | LE07_L2SP_195028_20190716_20200825_02_T1 |
|  |  | LE07_L2SP_195028_20190801_20200824_02_T1 |
|  | SS | LC08_L2SP_195028_20190926_20200825_02_T1 |
|  |  | LC08_L2SP_195028_20191012_20200825_02_T1 |
|  |  | LC08_L2SP_195028_20191028_20200825_02_T1 |
|  |  | LC08_L2SP_195028_20191113_20200825_02_T1 |
|  |  | LC08_L2SP_195028_20191129_20200825_02_T1 |
|  |  | LE07_L2SP_195028_20190918_20200824_02_T1 |
|  |  | LE07_L2SP_195028_20191105_20200824_02_T1 |
| 2020 | GS | LC08_L2SP_195028_20200624_20200824_02_T1 |
|  |  | LC08_L2SP_195028_20200710_20200912_02_T1 |
|  |  | LC08_L2SP_195028_20200726_20200908_02_T1 |
|  |  | LC08_L2SP_195028_20200811_20200918_02_T1 |
|  |  | LC08_L2SP_195028_20200827_20200906_02_T1 |
|  |  | LE07_L2SP_195028_20200616_20200823_02_T1 |
|  |  | LE07_L2SP_195028_20200702_20200908_02_T1 |
|  |  | LE07_L2SP_195028_20200718_20200904_02_T1 |
|  |  | LE07_L2SP_195028_20200819_20200914_02_T1 |
|  | SS | LC08_L2SP_195028_20200928_20201006_02_T1 |
|  |  | LC08_L2SP_195028_20201014_20201105_02_T1 |
|  |  | LC08_L2SP_195028_20201030_20201106_02_T1 |
|  |  | LC08_L2SP_195028_20201115_20210315_02_T1 |
|  |  | LE07_L2SP_195028_20201107_20201203_02_T1 |
|  |  | LE07_L2SP_195028_20201123_20201220_02_T1 |
| 2021 | GS | LC08_L2SP_195028_20210713_20210721_02_T1 |
|  |  | LC08_L2SP_195028_20210729_20210804_02_T1 |
|  |  | LC08_L2SP_195028_20210814_20210819_02_T1 |
|  |  | LC08_L2SP_195028_20210830_20210909_02_T1 |
|  |  | LE07_L2SP_195028_20210721_20210816_02_T1 |
|  |  | LE07_L2SP_195028_20210806_20210902_02_T1 |
|  |  | LE07_L2SP_195028_20210822_20210917_02_T1 |
|  | SS | LC08_L2SP_195028_20210915_20210925_02_T1 |
|  |  | LC08_L2SP_195028_20211017_20211026_02_T1 |
|  |  | LC08_L2SP_195028_20211102_20211109_02_T1 |
|  |  | LC08_L2SP_195028_20211118_20211125_02_T1 |
|  |  | LC09_L2SP_195028_20211106_20220119_02_T1 |
|  |  | LC09_L2SP_195028_20211106_20230507_02_T1 |
|  |  | LE07_L2SP_195028_20210923_20211019_02_T1 |
|  |  | LE07_L2SP_195028_20211009_20211104_02_T1 |
|  |  | LE07_L2SP_195028_20211025_20211121_02_T1 |
|  |  | LE07_L2SP_195028_20211126_20211223_02_T1 |
| 2022 | GS | LC08_L2SP_195028_20220716_20220726_02_T1 |
|  |  | LC08_L2SP_195028_20220801_20220806_02_T1 |
|  |  | LC09_L2SP_195028_20220708_20220710_02_T1 |
|  |  | LC09_L2SP_195028_20220708_20230408_02_T1 |
|  |  | LC09_L2SP_195028_20220724_20220726_02_T1 |
|  |  | LC09_L2SP_195028_20220724_20230406_02_T1 |
|  |  | LC09_L2SP_195028_20220809_20220811_02_T1 |
|  |  | LC09_L2SP_195028_20220809_20230403_02_T1 |
|  |  | LC09_L2SP_195028_20220825_20220830_02_T1 |
|  |  | LC09_L2SP_195028_20220825_20230401_02_T1 |
|  |  | LE07_L2SP_195028_20220626_20220828_02_T1 |
|  |  | LE07_L2SP_195028_20220713_20220903_02_T1 |
|  |  | LE07_L2SP_195028_20220730_20220903_02_T1 |
|  |  | LE07_L2SP_195028_20220816_20220911_02_T1 |
|  | SS | LC08_L2SP_195028_20220918_20220928_02_T1 |
|  |  | LC08_L2SP_195028_20221004_20221012_02_T1 |
|  |  | LC08_L2SP_195028_20221020_20221101_02_T1 |
|  |  | LC08_L2SP_195028_20221105_20221115_02_T1 |
|  |  | LC08_L2SP_195028_20221121_20221129_02_T1 |
|  |  | LC09_L2SP_195028_20220926_20220928_02_T1 |
|  |  | LC09_L2SP_195028_20221012_20221014_02_T1 |
|  |  | LC09_L2SP_195028_20221012_20230326_02_T1 |
|  |  | LC09_L2SP_195028_20221028_20221030_02_T1 |
|  |  | LC09_L2SP_195028_20221028_20230324_02_T1 |
|  |  | LE07_L2SP_195028_20220919_20221015_02_T1 |
|  |  | LE07_L2SP_195028_20221006_20221102_02_T1 |
| 2023 | GS | LC08_L2SP_195028_20230617_20230623_02_T1 |
|  |  | LC08_L2SP_195028_20230703_20230711_02_T1 |
|  |  | LC08_L2SP_195028_20230719_20230802_02_T1 |
|  |  | LC08_L2SP_195028_20230820_20230826_02_T1 |
|  |  | LC09_L2SP_195028_20230625_20230627_02_T1 |
|  |  | LC09_L2SP_195028_20230711_20230713_02_T1 |
|  |  | LC09_L2SP_195028_20230727_20230802_02_T1 |
|  |  | LC09_L2SP_195028_20230812_20230814_02_T1 |
|  |  | LE07_L2SP_195028_20230701_20230727_02_T1 |
|  |  | LE07_L2SP_195028_20230706_20230801_02_T1 |
|  |  | LE07_L2SP_195028_20230728_20230823_02_T1 |
|  |  | LE07_L2SP_195028_20230819_20230914_02_T1 |
|  |  | LE07_L2SP_195028_20230824_20231223_02_T1 |
|  | SS | LC08_L2SP_195028_20231007_20231011_02_T1 |
|  |  | LC08_L2SP_195028_20231108_20231117_02_T1 |
|  |  | LC08_L2SP_195028_20231124_20231129_02_T1 |
|  |  | LC09_L2SP_195028_20230929_20231002_02_T1 |
|  |  | LC09_L2SP_195028_20231015_20231016_02_T1 |
|  |  | LE07_L2SP_195028_20230920_20231016_02_T1 |
|  |  | LE07_L2SP_195028_20231012_20231107_02_T1 |
|  |  | LE07_L2SP_195028_20231017_20231112_02_T1 |
